# Phosphorylation of LAMP2A by p38 MAPK couples ER stress to chaperone-mediated autophagy

**DOI:** 10.1038/s41467-017-01609-x

**Published:** 2017-11-24

**Authors:** Wenming Li, Jinqiu Zhu, Juan Dou, Hua She, Kai Tao, Haidong Xu, Qian Yang, Zixu Mao

**Affiliations:** 1 Departments of Pharmacology and Neurology, Emory University School of Medicine, Atlanta, GA 30322 USA; 2 Department of Neurosurgery, Tangdu Hospital, The Fourth Military Medical University, Xi’an, Shaanxi 710038 China; 3 Department of Epidemiology and Environmental Health, University at Buffalo, Buffalo, NY 14214 USA

## Abstract

Endoplasmic reticulum (ER) and lysosomes coordinate a network of key cellular processes including unfolded protein response (UPR) and autophagy in response to stress. How ER stress is signaled to lysosomes remains elusive. Here we find that ER disturbance activates chaperone-mediated autophagy (CMA). ER stressors lead to a PERK-dependent activation and recruitment of MKK4 to lysosomes, activating p38 MAPK at lysosomes. Lysosomal p38 MAPK directly phosphorylates the CMA receptor LAMP2A at T211 and T213, which causes its membrane accumulation and active conformational change, activating CMA. Loss of ER stress-induced CMA activation sensitizes cells to ER stress-induced death. Neurotoxins associated with Parkinson’s disease fully engages ER-p38 MAPK–CMA pathway in the mouse brain and uncoupling it results in a greater loss of SNc dopaminergic neurons. This work identifies the coupling of ER and CMA as a critical regulatory axis fundamental for physiological and pathological stress response.

## Abstract (web summary)

The endoplasmic reticulum (ER) and lysosome are central to cellular stress responses, but it is unclear how ER stress is signaled to lysosomes. Here the authors show that ER stress activates chaperone-mediated autophagy (CMA) via direct phosphorylation of the CMA receptor LAMP2A by the lysosomal p38 MAPK.

## Introduction

Pathologic stress induces loss of specific populations of neurons, which underlies the pathological process of many neurodegenerative diseases^1, 2^. ER and lysosomes are two primary organelles in neurons responsible for processing stress signals and executing a range of proper cellular responses. ER disturbance triggers a critical process known as unfolded protein response (UPR). This response is characterized by reduced loading of proteins requiring folding and processing in the ER lumen and increased capacity for protein folding, processing, and ER-associated degradation (ERAD). Several distinct pathways regulate these responses. These include transmembrane ER stress sensors, PERK (double-stranded RNA-activated protein kinase (PKR)-like ER kinase), ATF6 (activating transcription factor 6), and IRE1α (the inositol-requiring enzyme 1α). The initial UPR promotes an adaptive mechanism to restore ER homeostasis and maintain cellular viability^3^; but when ER stress becomes insurmountable, UPR also leads to apoptosis^3^. Cellular stress also activates macroautophagy (MA, also referred to autophagy), a process involving degradation of cellular components by lysosomes^4^. Chaperone-mediated autophagy (CMA) selectively degrades individual cytosolic proteins^5, 6^. This process does not require the formation of vacuole and is tightly controlled by two key CMA regulators, chaperone Hsc70 and the receptor, lysosome-associated membrane protein 2 A (LAMP2A). Hsc70 binds to substrate proteins, which contain a KFERQ-like motif, and target them to LAMP2A on lysosomes for degradation. Little is known on how LAMP2A is regulated. Accumulating evidence indicates that dysfunction of autophagy including CMA plays an important role in neurodegeneration including Parkinson’s disease (PD)^7–9^. Although dysfunction of both ER and CMA has been observed in postmortem brains^10, 11^, it is completely unclear whether ER stress and CMA are functionally linked.

In this study, we demonstrate that ER stress is coupled to CMA. This coupling requires PERK-dependent activation and association of MKK4 with lysosomes and activation of a lysosomal pool of p38 MPAK. The activated lysosomal p38 MAPK then directly phosphorylates LAMP2A, causing its accumulation and oligomerization on the lysosomal membrane and activating CMA. We term this coupling ERICA for ER stress-induced CMA. Engaging ERICA is functionally required for maintaining cellular homeostasis and protecting cells from initial stress while uncoupling it is associated with increased neuronal death in vivo in a neurotoxin-induced model of PD.

## Results

### ER stress activates CMA by increasing CMA receptor LAMP2A

**Fig. 1:**
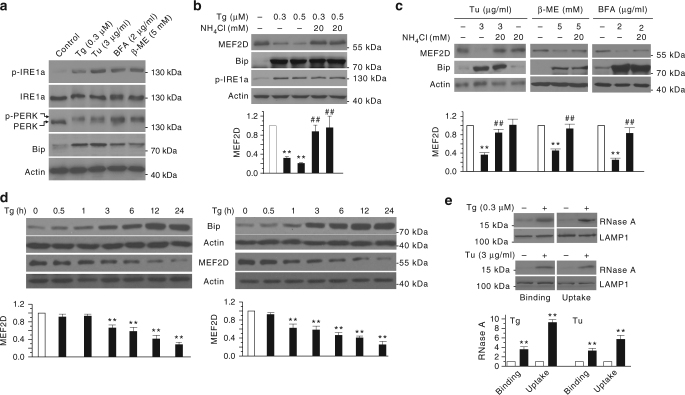
ER stress activates CMA. **a** ER stressors induce UPR in SN4741 cells. SN4741 cells were treated for 5 h with Tg (Thapsigargin), Tu (Tunicamycin), β-mercaptoethanol (β-ME), or brefeldin A (BFA). Various UPR markers were determined by immunoblotting (activated PERK has reduced mobility due to phosphorylation). **b** Inhibition of lysosomal activity attenuates Tg-induced MEF2D degradation. SN4741 cells were exposed to Tg with or without NH_4_Cl for 12 h. Protein levels of MEF2D, Bip, and phospho-inositol-requiring enzyme 1 (p-IRE1α) were determined. **c** Multiple ER stressors induce lysosomal dependent degradation of MEF2D. SN4741 cells were exposed to Tu, β-ME, BFA with or without NH_4_Cl for 12 h. **d** ER stress induces time-dependent degradation of MEF2D. Tg and Tu caused an increase of Bip level and a reduction of MEF2D level in SN4741 cells in a time-dependent manner. **e** ER stress enhances CMA activity in SN4741 cells. Lysosome binding (left panel) and uptake (right panel) of a commercially purified CMA substrate RNase A were determined after incubation with lysosomes isolated from SN4741 cells treated with or without Tg 0.3 μM or Tu 3 µg/ml for 12 h. LAMP1 was used as the lysosomal loading control. Bottom panels of **b** to **e** show the quantification of CMA substrate MEF2D or RNase A relative to untreated cells (*n* = 3 of independent experiments). All values are mean ± s.d. (ANOVA with Turkey test for **b**, **c** and **d**, unpaired two-tailed *t* test for **e**; **p < 0.005 vs. control and ^##^ *p* < 0.005 vs. Tg or Tu alone)

Because both UPR and CMA are involved in disposing proteins upon stress, we investigated the possibility that these two key protein quality control processes may be functionally related. For this, we treated SN4741 cells, a mouse midbrain dopaminergic progenitor cell line, with four ER stressors known to induce UPR, including Ca^2+^pump inhibitor thapsigargin (Tg), N-glycosylation suppresser tunicamycin (Tu), reducing agent 2-mercaptoethanol (β-ME), and ER-Golgi protein transport inhibitor Brefeldin A (BFA). These treatments caused a robust ER stress as indicated by a clear elevation of the levels of three key ER stress sensors, phospho-IRE1α, phospho-PERK, and BiP/GRP78 (Fig. 1a). Previous studies have shown that the level of transcription factor myocyte enhancer 2D (MEF2D), a known CMA substrate, is very sensitive to stress in neurons and SN4741 cells^12^. Examination of MEF2D showed that all four ER stress inducers cause a clear decrease of MEF2D level and NH_4_Cl attenuated ER stress-induced reduction of MEF2D protein (Fig. 1b, c). 3-MA and MG132, well-known MA and proteasome inhibitors, respectively, had no effect on Tg-induced and Tu-induced MEF2D reduction (Supplementary Fig. 1a), consistent with the previous findings that MEF2D is preferentially degraded by CMA, but not MA and ubiquitin-proteasome system^12, 13^. Detailed time course analysis indicated that the reduction of MEF2D level parallels closely with the induction of ER stress (Fig. 1d), suggesting a more direct and robust mechanism contributing to MEF2D degradation. To rule out the possibility that ER stress-induced decrease of MEF2D is caused by PERK-mediated inhibition of mRNA and translation, we knocked down PERK in SN4741 cells and found that Tg does not significantly affect mRNA expression of MEF2D and knockdown of PERK does not affect MEF2D mRNA and protein expressions under both basal and Tg treatment condition (Supplementary Fig. 1b). We investigated the possibility that ER stress may regulate CMA using Tg and Tu as our primary model agents for the subsequent studies. Following Tg-induced and Tu-induced ER stress, we prepared highly purified lysosomes (Supplementary Fig. 1c) and directly measured CMA activity by lysosomal binding and uptake assays using two well-established CMA substrates ribonuclease A (RNase A)^14–17^ and MEF2D^12, 18^. With equal loading of lysosomal marker LAMP1, Tg and Tu markedly increased the binding and uptake of RNase A and MEF2D by lysosomes (Fig. 1e; Supplementary Fig. 1d). These findings indicate that ER stress causes a robust activation of CMA.

**Fig. 2:**
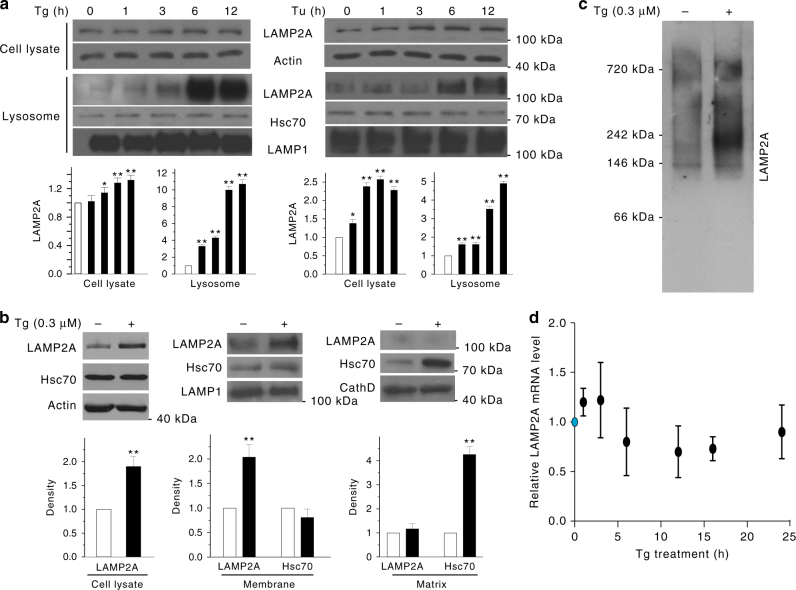
ER stress increases the lysosomal level of CMA regulator LAMP2A. **a** ER stress increases the level of CMA regulator LAMP2A. Total or purified lysosomal lysates from SN4741 cells treated with Tg or Tu for 0–12 h were blotted as indicated. **b** ER stress increases LAMP2A on lysosomal membrane. Whole lysosomes, lysosomal membrane, and matrix were prepared from SN4741 cells treated with Tg for 12 h and blotted for LAMP2A and Hsc70. **c** ER stress induces LAMP2A oligomerization. Lysosomes purified from Tg-treated SN4741 cells were subjected to native continuous gel electrophoresis and immunoblotted for LAMP2A. **d** ER stress does not affect the level of LAMP2A mRNA. SN4741 cells were treated with Tg (0.3 μM) for different time periods. The levels of LAMP2A mRNA were determined by qRT-PCR. Data are shown as mean ± s.d. *n* = 3 × 4 repeats (One-way ANOVA with Turkey, *p* > 0.05). Bottom panels of **a** and **b** show quantification of LAMP2A and LAMP2A/Hsc70 relative to basal condition (*n* = 3 of independent experiments). All values are mean ± s.d. (ANOVA with Turkey test for **a**, unpaired two-tailed *t* test for **b**; *p < 0.05 or **p < 0.005 vs. control)

CMA activity is largely controlled by two key regulators, chaperone protein Hsc70 and the rate limiting lysosomal membrane receptor LAMP2A^5^. We determined their levels following Tg-induced or Tu-induced ER stress. Treatment of SN4741 cells with Tg or Tu significantly increased the level of LAMP2A in purified lysosomes (Fig. 2a). Analysis of the sub-lysosomal fractions showed that Tg treatment markedly increased LAMP2A in the lysosomal membrane and Hsc70 in the matrix (Fig. 2b). Activation of CMA is known to be associated with oligomerization of LAMP2A^19^. Non-reducing native gel analysis of the purified lysosomal membrane revealed that Tg greatly increases the level of LAMP2A migrating as well-resolved higher-molecular-mass complexes (>200 kDa) (Fig. 2c). Tg treatment did not significantly alter the level of LAMP2A mRNA measured by RT-qPCR (Fig. 2d). Furthermore, the protein synthesis inhibitor cycloheximide did not abolish the increase of LAMP2A induced by Tg and Tu in either whole cell lysates or purified lysosomes, but it markedly attenuated the level of Bip following Tg and Tu treatment (Supplementary Fig. 2). Thus, these results together suggest strongly that ER stress activates CMA mainly through altering the level and conformation of LAMP2A protein on the lysosomal membrane.

### p38 mediates ER stress-induced CMA by phosphorylating LAMP2A

**Fig. 3:**
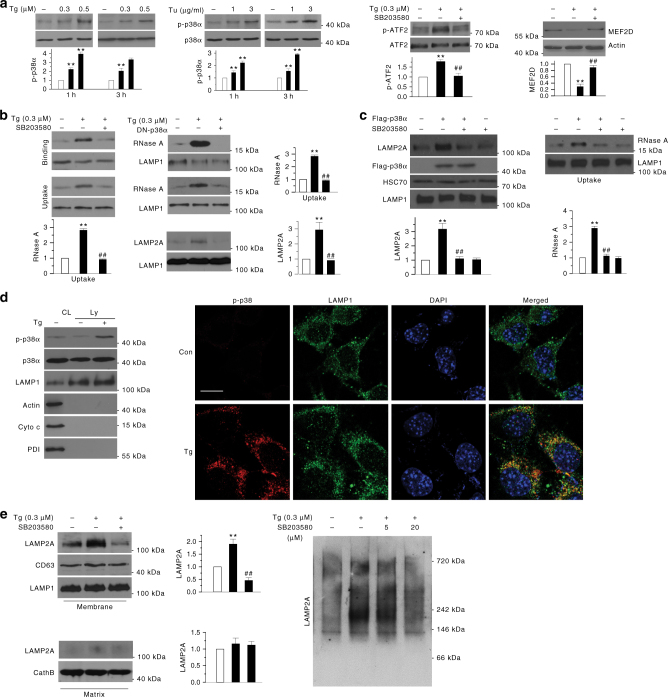
p38 MAPK mediates ER stress-induced CMA. **a** ER stress activates p38α MAPK. Phospho-p38α MAPK (p-p38α) levels were increased in SN4741 cells after exposure to Tg or Tu for 1 h or 3 h (left and middle sets of panels). Tg (3 h) increased the level of p38 MAPK substrate p-ATF2 (activating transcription factor 2) in SN4741 cells and Tg (12 h) decreased the level of MEF2D (right set of panels). **b** Inhibition of p38 MAPK prevents ER stress-induced CMA activation. CMA activity was determined by lysosome binding (top left) and uptake (bottom left) assays using a commercially purified CMA substrate RNase A following incubation of lysosomes purified SN4741 cells exposed to Tg with or without SB203580 for 12 h. SN4741 cells were transfected with dominant-negative p38α MAPK (DN-p38α) or control vector, treated with Tg as described above, and assayed for RNase A binding and uptake (right top and middle panels). Bottom shows the levels of LAMP2A in lysosomes under various conditions. **c** Increasing p38 MAPK activity is sufficient to activate CMA. Lysosomes isolated from SN4741 cells transfected with constructs as indicated were blotted for LAMP2A and Hsc70 (left) and tested for RNase A uptake (right). **d** ER stress increases the level of p-p38α MAPK on lysosomes. Cytosolic fraction (CL) and purified lysosomes (Ly) isolated from SN4741 after Tg treatment (0.3 μM, 2 h) were blotted for various proteins as indicated (left panel: LAMP1, lysosome marker; PDI, ER marker; cytochrome C (Cyto C), mitochondrial marker) or were analyzed by immunocytochemistry (right panel. Scale bar = 10 µm). **e** SB203580 attenuates ER stress-induced increase of LAMP2A on lysosomal membrane. SN4741 cells were exposed to Tg for 12 h with or without 1 h SB203580 pretreatment. Lysosomal membrane and matrix fractions were analyzed for LAMP2A (left). Purified lysosomes were blotted for oligomerized LAMP2A (right). Quantifications are shown for **a**, **b**, **c**, and **e** (*n* = 3 (ANOVA with Turkey test); **p < 0.005 vs. control and ##*p* < 0.005 vs. corresponding challenge alone)

We studied the molecular mechanism(s) underlying ER stress-induced activation of CMA. p38 MAPK is a key kinase activated by many cellular stress conditions including ER stress^20, 21^. Our analysis showed that Tg and Tu treatment increases p38 MAPK phosphorylation at T180/Y182 without changing the total level of p38α MAPK (the major isoform of p38 MAPK in SN4741) (Fig. 3a, left), confirming that ER stress activates the p38α MAPK pathway. To test whether p38α MAPK plays a role in ER stress-induced activation of CMA, we treated SN4741 cells with Tg and SB203580, a widely used pharmacological inhibitor of p38α MAPK, and showed that SB203580 reduces the level of phospho ATF2, a known p38α MAPK substrate, and protects MEF2D from Tg-induced degradation (Fig. 3a, right). SB203580 did not alter Tg-induced increase of either Bip or LC3II (Supplementary Fig. 3a, b), indicating that the effect of SB203580 on the MEF2D level is likely through the inhibition of CMA, but not due to a non-specific blockade of ER stress response. To substantiate this, we co-treated the cells with Tg and SB203580, purified the lysosomes, and showed that SB203580 significantly reduces Tg-induced activation of CMA by lysosomal binding and uptake assays (Fig. 3b, left; Supplementary Fig. 1d). To further strengthen these findings, we blocked endogenous p38 MAPK activity by overexpressing a dominant-negative p38α MAPK mutant and showed that this mutant greatly attenuates Tg-induced increase of LAMP2A and CMA activity using purified lysosomes (Fig. 3b, right). To test whether activation of p38α MAPK is sufficient for inducing CMA, we overexpressed p38α MAPK with or without SB203580 in HEK293 cells, isolated lysosomes, and showed that overexpression of p38α MAPK significantly increases the lysosomal LAMP2A and CMA activity (Fig. 3c). SB203580 reversed the p38α MAPK-induced changes, suggesting that high level of p38α MAPK activity is sufficient to activate CMA. Interestingly, analysis of the purified lysosomes showed that a fraction of p38α is localized to the lysosomes under both basal and ER stress conditions (Fig. 3d, left). Although Tg treatment did not cause a significant change in the level of total p38α MAPK associated with the lysosomes, ER stress did significantly increase the level of p-p38α MAPK on the lysosomes. Consistent with this, immunocytochemistry analysis showed that exposure to Tg increases the p-p38α MAPK signal that co-localizes with the lysosomes (Fig. 3d, right). Inhibition of p38α MAPK by SB203580 attenuated Tg-induced activation of p38α MAPK (Supplementary Fig. 3c) and the levels of total as well as oligomerized LAMP2A associated with the lysosomes (Fig. 3e).

**Fig. 4:**
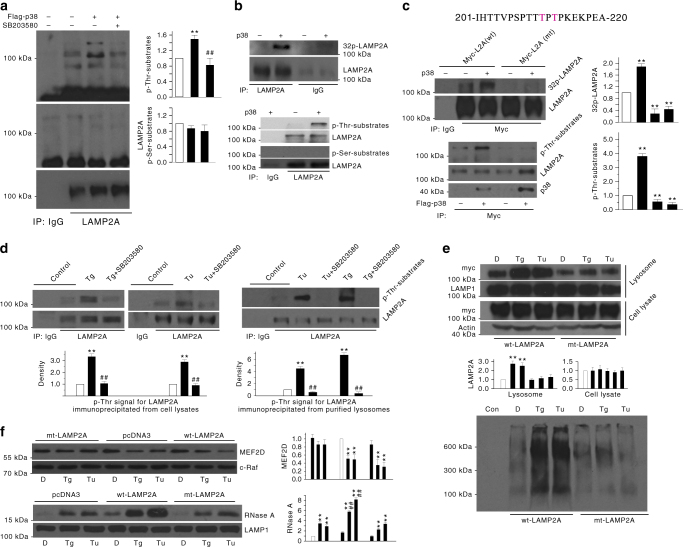
p38 MAPK directly phosphorylates LAMP2A. **a** High level of p38 MAPK leads to LAMP2A phosphorylation in SN4741 cells. SN4741 cells were transfected with p38 MAPK for 40 h with or without SB203580 (20 μM), and proteins were collected for IP with anti-LAMP2A and IB with antibodies to p-Thr-substrates, p-Ser-substrates, or LAMP2A. **b** p38 MAPK directly phosphorylates human LAMP2A in vitro. Endogenous LAMP2A proteins were immunoprecipitated from HEK293 cells, and the precipitates were incubated with purified p38 MAPK in vitro kinase assay with^32^P-ATP or cold ATP following by anti-p-Thr or p-Ser blotting. (**c**) T211 and T213 are required for p38 MAPK-mediated phosphorylation of LAMP2A. HEK293 cells were transfected with myc-LAMP2A (wt) or (mt, T211A and T213A) for 40 h, and proteins were collected for IP with anti-myc for p38 MAPK in vitro kinase assay (top left); and SN4741 cells were transfected with LAMP2A (wt), or LAMP2A(mt) with Flag-p38 MAPK for 40 h and proteins were collected for IP with anti-myc and IB with anti-p-Thr-substrate antibody. **d** ER stress induces LAMP2A phosphorylation in a p38 MAPK-dependent manner. SN4741 cells were treated with Tg (0.3 μM) or Tu (3 μg/ml) for 2 or 1 h, respectively. Total (top) or lysosomal (bottom) proteins were extracted from the cells for IP with LAMP2A antibody and IB with anti-p-Thr-substrate antibody. **e** ER stress increases lysosomal LAMP2A and its oligomerization in a phosphorylation-dependent manner. SN4741 cells were transfected with myc-wt-LAMP2A or myc-mt-LAMP2A (T211A and T213A) for 24 h and then were treated with Tg (0.3 μM) or Tu (3 μg/ml) for 12 h. Lysosomal proteins were blotted following SDS-PAGE (top) or blue negative gel (bottom) with anti-myc antibody. **f** ER stress increases CMA activity in an LAMP2A phosphorylation-dependent manner. SN4741 cells were transfected with pcDNA3, wt-LAMP2A, or mt-LAMP2A (T211A and T213A) for 24 h and then were treated with Tg (0.3 μM) or Tu (3 μg/ml) for 12 h. Cytoplasmic proteins were blotted with an MEF2D antibody (top), and intact lysosomes were assessed in uptake assay (bottom). Quantifications are shown for **a**, **c**, **d**, **e**, and **f** (*n* = 3. All values are mean ± s.d. (ANOVA with Turkey test). **p < 0.005 vs. control and ##*p* < 0.005 vs. corresponding challenge alone)

Since p38 MAPK is required for ER stress-induced regulation of LAMP2A and CMA, we tested the possibility that p38 MAPK may directly phosphorylate and regulate LAMP2A. First, we carried out immunoprecipitation and established that the endogenous p38 MAPK and endogenous LAMP2A associate with each other in SN4741 cells under basal condition. Furthermore, Tg treatment enhanced their binding (Supplementary Fig. 4a). We then transfected p38α MAPK into SN4741 cells, immunoprecipitated the endogenous LAMP2A, and blotted with antibodies to either phosphorylated Thr or Ser residues. This analysis revealed that high p38 MAPK activity leads to a robust phosphorylation of LAMP2A at Thr residues and that this is abolished by SB203580 (Fig. 4a). For a direct phosphorylation, we incubated the purified p38α MAPK with either overexpressed mouse LAMP2A or endogenous LAMP2A precipitated from HEK293 cells, and showed that p38 MAPK directly phosphorylates LAMP2A of mouse and human origin mainly at Thr sites (Supplementary Fig. 4b and Fig. 4b). Using the GPS3.0 software, threonines at position 211 and 213 were predicted as putative phosphorylation sites for p38 MAPK (Supplementary Table 1 and Fig. 4c top). We mutated threonines 211 and 213 to alanines and showed that mutation of threonines 211 and 213 completely abolishes phosphorylation of LAMP2A by p38 MAPK in in vitro kinase assay (Fig. 4c, middle) and anti-phospho Thr assay (Fig. 4c bottom and Supplementary Fig. 4c). To test if these two sites are indeed critical in sensing ER stress, we first showed that both Tg and Tu induce a robust Thr phosphorylation of protein running at LAMP2A position in a time-dependent manner (Supplementary Fig. 4d). Phospho Thr blotting of immunoprecipitated LAMP2A showed that ER stress increases LAMP2A phosphorylation at Thr residues in a p38 MAPK-dependent manner in both whole cellular lysates (Fig. 4d, left) and lysosomal fraction (Fig. 4d, right). Tg and Tu treatment increased the lysosomal level of total and oligomerized wt-LAMP2A overexpressed in cells; however, Tg and Tu failed to alter the lysosomal level of LAMP2A Thr211/213Ala mutant (Fig. 4e). Similarly, overexpression of wt-LAMP2A enhanced Tg-induced or Tu-induced increase of CMA and decrease of MEF2D. Mutation of Thr at 211 and 213 to Ala rendered LAMP2A incapable of responding to Tg or Tu and enhancing CMA activity (Fig. 4f).

### PERK/MKK4/p38 mediates ER stress-induced activation of CMA

**Fig. 5:**
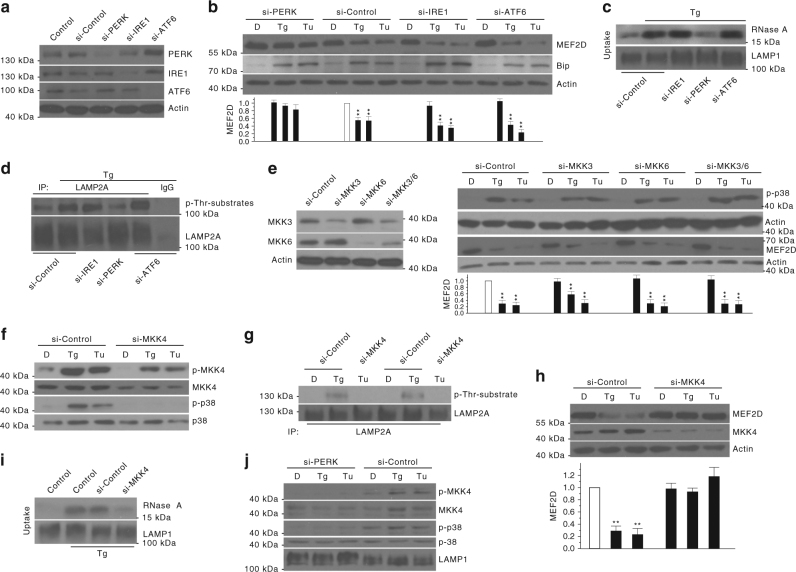
PERK and MKK4 are required to activate the p38 MAPK–CMA pathway under ER stress. **a** siRNAs effectively knockdown PERK, IRE1, and ATF6. SN4741 cells were transfected with siRNAs for 72 h. **b**, **c** Knockdown of PERK abolishes the activation of CMA by ER stress. SN4741 cells were transfected with siRNAs for 60 h, then treated with Tg (0.3 μM) or Tu (3 μg/ml) for 12 h, and assessed for endogenous CMA substrate MEF2D level **b** and RNase A uptake **c**. **d** Knockdown of PERK reduces p-LAMP2A. SN4741 cells were transfected siRNAs for 69 h, and then treated with Tg for 2 h. **e** MKK3 and 6 are not involved in ER stress-induced CMA. SN4741 cells were transfected with si-Control, si-MKK3, si-MKK6, and si-MKK3 as well as 6 (MKK3/6) for 72 h. Total proteins were blotted with MKK3 and MKK6 antibodies (left). SN4741 cells were transfected for 70 h and exposed to Tg or Tu for 2 h for p-p38 MAPK (top right) or transfected for 60 h and exposed to Tg or Tu for 12 h for MEF2D (bottom right). **f**, **g** Knockdown of MKK4 attenuates activation of p38 MAPK and phosphorylation of LAMP2A induced by ER stress. SN4741 cells were transfected with si-Control and si-MKK4 for 70 h, and then treated with Tg or Tu for 2 h (**f**) or transfected for 69 h and stressed for 3 h (**g**). **h**, **i** Knockdown of MKK4 reverses the reduction of MEF2D induced by ER stress. SN4741 cells were transfected with si-Control and si-MKK4 for 60 h, treated with Tg or Tu for 12 h, and assessed for MEF2D level (**h**) and RNase A uptake (**i**). **j** Knockdown of PERK reverses the activation of MKK4 and p38 MAPK by ER stress. SN4741 cells were transfected with si-Control and si-PERK for 70 h, and then treated with Tg or Tu for 2 h. Quantifications are shown for **b**, **e**, and **h** (*n* = 3. All values are s.d. (one-way ANOVA with Turkey test). **p < 0.005 vs. control and ^##^ *p* < 0.005 vs. corresponding challenge alone)

To determine which UPR sensor(s) may be involved in mediating ER stress-induced activation of CMA, we transfected SN4741 cells with siRNAs for UPR transducers PERK, IRE1α, and ATF6 individually and showed that all three siRNAs can efficiently and specifically reduce the level of their corresponding target protein (Fig. 5a), and the knockdown of PERK and IRE1α significantly block ATF4/CHOP expression and XBP1 splicing induced by Tg, respectively (Supplementary Fig. 5a, b). We then exposed cells after siRNA transfection to ER stress and analyzed CMA. Our analysis showed that knockdown of PERK, but not IRE1α and ATF6, significantly suppressed Tg-induced and Tu-induced decrease of CMA substrate MEF2D (Fig. 3b). Furthermore, knockdown of PERK did not affect the MEF2D mRNA level by qRT-PCR under basal and Tg treatment conditions (Supplementary Fig. 1b). Consistently, knockdown of PERK inhibited Tg-induced increase of CMA activity by lysosomal uptake assay (Fig. 5c) and abolished Tg-induced phosphorylation of lysosomal LAMP2A (Fig. 5d). MKK3/6 and ASK1 are classical upstream activators of p38 MAPK^22, 23^. Although their knockdowns significantly attenuated their protein expression (Fig. 5e left and Supplementary Fig. 5c top left) and ASK1 knockdown blocked Tg-induced and Tu-induced JNK activation (Supplementary Fig. 5c, middle left), these knockdowns failed to prevent ER stress-induced activation of p38 MAPK and reduction of MEF2D (Fig. 5e right and Supplementary Fig. 5c right). ER stress greatly activated MKK4, another upstream p38 MAPK regulator, in SN4741 cells, and knockdown of MKK4 attenuated Tg-induced or Tu-induced phosphorylation of p38 MAPK in our model system (Fig. 5f). More importantly, knockdown of MKK4 also significantly reversed Tg-induced or Tu-induced phosphorylation of lysosomal LAMP2A (Fig. 5g), Tg-induced or Tu-induced reduction of CMA substrate MEF2D (Fig. 5h), and Tg-induced lysosomal uptake of CMA substrate RNase A (Fig. 5i). Interestingly, analysis of the highly purified lysosomal fraction showed that ER stress significantly increases the level of both total and phosphorylated MKK4 in lysosomes. This lysosomal association of MKK4 paralleled closely with the increased phosphorylation of lysosomal p38 MAPK (Fig. 5j). Knockdown of PERK completely abolished ER stress-induced increase in total and phosphorylated MKK4 and phosphorylated p38 MAPK in lysosomes (Fig. 5j). Consistent with these, PERK inhibitor GSK2606414, but not IRE1 inhibitor 4µ8c, significantly reduced the levels of p-MKK4, p-p38 MAPK, phosphorylation and oligomerization of LAMP2A, and CMA activity induced by Tg in SN4741 cells (Supplementary Fig. 5e). These findings indicate that PERK-lysosomal association of activated MKK4 stimulates lysosomal p38 MAPK to mediate ER stress-induced activation of CMA.

### Lysosomal p38 activation protects cells against ER stress

**Fig. 6:**
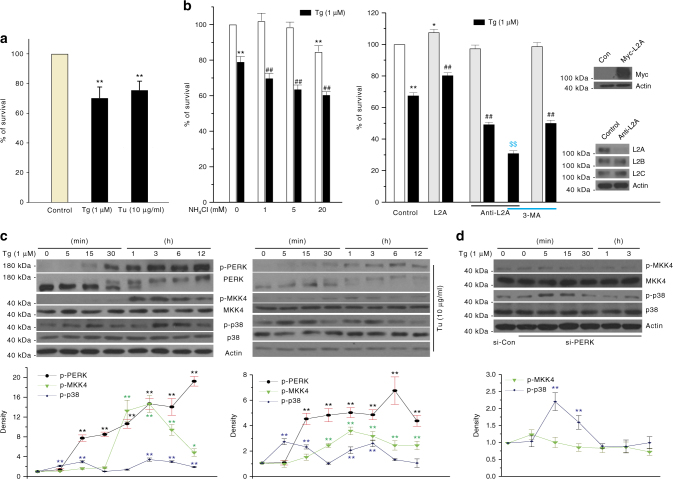
ER stress-induced CMA is cellular protective. **a** ER stress induces cell death. SN4741 cells were exposed to Tg or Tu for 24 h. Cell viability was measured by MTT assay. **b** Blocking lysosomal function aggravates ER stress-induced cell death. SN4741 cells were treated with Tg with or without NH_4_Cl at indicated concentrations for 24 h (left panel). After 24 h transfection of HA-LAMP2A (L2A) or 48 h anti-sense LAMP2A (Anti-L2A), SN4741 cells were treated with Tg alone or with 3-MA (5 mM) for 24 h. Cell viability was measured by MTT assay. Inlet shows the levels of LAMP2A overexpression or knockdown and the levels of LAMP2B and LAMP2C. Values in **a** and **b** are mean ± s.e.m, (*n* = 3 × 4 replicates of independent experiments (ANOVA with Turkey test); *p < 0.05 or **p < 0.005 vs. control, ^##^ *p* < 0.05 vs. Tg alone group, ^$$^ *p* < 0.005 vs. anti-L2A with Tg group). **c** ER stress induces a biphasic activation of p38 MAPK. SN4741 cells were treated with Tg or Tu at indicated time periods. Total proteins were blotted as indicated. **d** Knockdown of PERK specifically blocks the second wave of p38 MAPK activation. SN4741 cells transfected with control and si-PERK were treated with Tg for various periods of time. Quantifications are shown at **c** and **d** bottom panels (Values are mean ± s.d. *n* = 3 (ANOVA with Turkey test); **p < 0.005 vs. control)

**Fig. 7:**
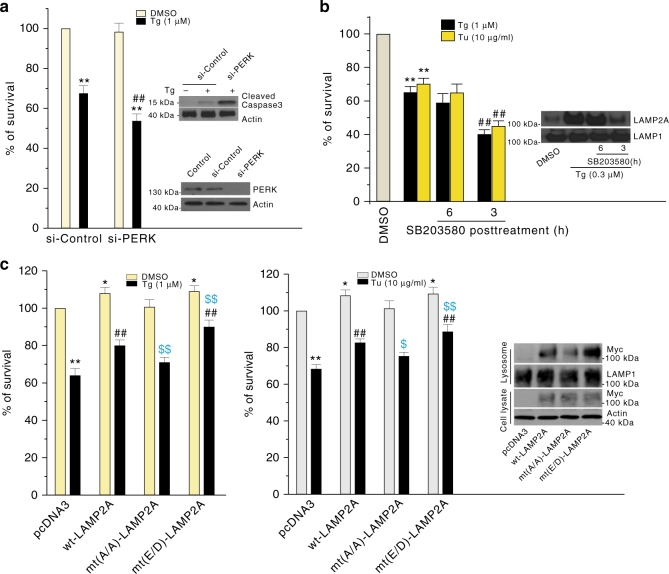
PERK-p38–MAPK–phosphor–LAMP2A pathway mediates cellular protection. **a** Knockdown of PERK aggravates Tg-induced cell death in SN4741 cells. SN4741 cells were transfected with si-Control and si-PERK for 48 h and then treated with Tg (1 μM) for 24 h. The cell viability was measured by WST-1 assay and data were expressed as mean ± s.e.m. (*n* = 3 × 4 replicates (ANOVA with Turkey test); **p < 0.005 vs. si-Control, and ^##^ *p* < 0.005 vs. si-Control with Tg group). Total proteins were blotted for cleaved caspase 3 (top right) or PERK (bottom right). **b** The effect of p38 MAPK inhibition on ER stress-induced cell death is time-dependent. SN4741 cells were exposed to Tg and Tu for 3 or 6 h and then co-incubated with SB203580 for a total of 24 h Tg or Tu treatment. Cell viability was measured by WST-1 assay and data were expressed as mean ± s.e.m. (*n* = 3 × 4 replicates of independent experiments (ANOVA with Turkey test); **p < 0.005 vs. control, ^#^ *p* < 0.05 and ^##^ *p* < 0.005 vs. Tg or Tu alone group). Right panel shows that the increase of lysosomal LAMP2A induced by Tg can be abolished by SB203580 3 h, but not 6 h post Tg stress. **c** LAMP2A phosphorylation is necessary for protecting cells against ER stress. SN4741 cells were transfected with pcDNA3, myc-wt-LAMP2, mt (A/A)-LAMP2A (T211A and T213A), and mt (E/D)-LAMP2A (T211E and T213D) for 24 h, exposed to Tg or Tu for another 24 h, and assessed for viability by WST-1 assay. Data were expressed as mean ± s.e.m. (*n* = 3 × 4 replicates of independent experiments (ANOVA with Turkey test). *p < 0.05 or **p < 0.005 vs. control, ^##^ *p* < 0.005 vs. Tg or Tu alone group, ^$^ *p* < 0.05 and ^$$^ *p* < 0.01 vs. wt-LAMP2A with Tg or Tu alone group). Right panel shows the lysosomal levels of the overexpressed LAMP2A

ER stress modulates cellular viability. The initial cellular response to UPR is known to alleviate ER stress, re-establish ER homeostasis, and promote survival. But prolonged and severe ER stress induces apoptosis^24^. Prolonged Tg or Tu treatment clearly reduced the viability of SN4741 cells (Fig. 6a). The loss of cellular viability was significantly increased when cells were treated with NH_4_Cl together with Tg (Fig. 6b, left). Importantly, increasing or decreasing LAMP2A level by either overexpression of wt-LAMP2A or its anti-sense construct protected SN4741 cells from or sensitized them to ER stress-induced death, respectively (Fig. 6b, right). Moreover, inhibition of MA by 3-MA further increased cell death induced by a combination of Tg and LAMP2A knockdown (Fig. 6b, right). Thus, these findings indicate strongly that activation of CMA independent of MA following ER stress constitutes a critical part of the initial protective response. Detailed time course analysis revealed that ER stress clearly induces a biphasic activation of p38 MAPK. Interestingly, only the second (1–3 h post-ER stress), but not the first phase (5–15 min post-ER stress), of p38 MAPK activation was preceded by the activation of MKK4 (Fig. 6c) and kinetically correlated closely with CMA activity (Fig. 1 and Supplementary Fig. 4d). Knockdown of PERK did not affect the first phase, but greatly attenuated the second phase of p38 MAPK activation following ER stress (Fig. 6d). In addition, loss of PERK activity sensitized SN4741 cells to ER stress-induced death (Fig. 7a). Similarly, inhibition of the second p38 MAPK peak 3 h post initiation of ER stress by SB203580 correlated with a considerably higher level of CHOP and cleaved caspase 3 (Supplementary Fig. 6a), nuclear condensation by Hoechst staining (Supplementary Fig. 6b), and cell death measured by MTT (Fig. 7b). In contrast, SB203580 added 6 h post-ER stress did not significantly worsen cellular death (Fig. 7b). SB203580 alone did not affect the expression of cleaved caspase 3 and viability of SN4741 cells (Supplementary Fig. 6a–c). To further delineate the role of CHOP and CMA in ER stress-induced death^25, 26^, we modulated the levels of CHOP or LAMP2A by overexpression or anti-sense approaches and measured their effects on CMA activity, level of MEF2D, and levels of LAMP2A or CHOP, respectively, following Tg. These analyses showed that altering CHOP level does not affect CMA (Supplementary Fig. 6d). Interestingly, enhancing LAMP2A reduced Tg-induced increase of CHOP while knocking down LAMP2A had the opposite effect on CHOP (Supplementary Fig. 6e). Thus, CHOP is responsive to the level of LAMP2A and functions downstream of CMA in ER stress response. To establish the role of phosphorylation of LAMP2A in CMA-mediated protection, we overexpressed wt-, phosphorylation-resistant mt (A/A)-, and phosphorylation mimic mt (E/D)-LAMP2A, and then exposed cells to Tg or Tu. The viability analysis showed that both wt- and mt (E/D)-LAMP2A, but not mt (A/A)-LAMP2A offer cells significant protection against Tg-induced or Tu-induced death (Fig. 7c, left and middle). Correlated with this, overexpression of wt- and mt (E/D)-LAMP2A led to significantly higher level of lysosomal accumulation of LAMP2A than mt (A/A)-LAMP2A, although their overall expression levels in cells were comparable (Fig. 7c, right).

### ER stress engages p38-LAMP2A–CMA process in vivo

**Fig. 8:**
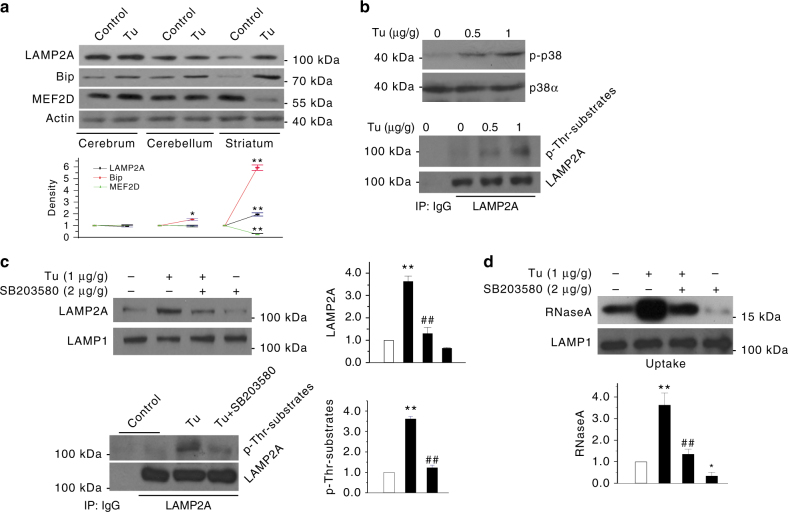
ER stress induces CMA activation in vivo. **a** Tu induces ER stress in mouse brain. Mice were injected (i.p.) with Tu (1 μg/g body weight), and then killed 2 days later for analysis (*n* = 12). Levels of LAMP2A, Bip, and MEF2D in various brain regions of mice were determined by western blotting. **b** ER stress induces p-LAMP2A on striatal lysosomes in a p38 MAPK-dependent manner. Mice were treated with Tu (0.5 or 1 μg/g body weight) alone or together with SB203580 for 24 h (*n* = 9). Lysosomes isolated from striatal tissue were blotted for p-p38 MAPK (Left), and for IP with LAMP2A and IB with p-Thr-substrate antibody (Right and bottom). **c**, **d** ER stress increases both LAMP2A level and CMA activity of lysosomes isolated from the striatum. Mice were injected (i.p.) as described in (**a**) with or without co-injection of SB203580 and then killed after 24 h (*n* = 9). Lysosomes isolated from the striatal tissue were analyzed for LAMP2A (**c**) or analyzed for CMA activity by uptake assay (**d**). Quantifications are shown for **a** to **d** (*n* = 6. All values are mean ± s.d. (unpaired two-tailed *t* test for LAMP2A/Bip/MEF2D, ANOVA with Turkey for others). *p < 0.05 or **p < 0.005 vs. control and ^##^ *p* < 0.005 vs. 6-OHDA)

To establish the ER stress-p38 MAPK–LAMP2A–CMA pathway in vivo, we treated 16-week-old mice with ER stressor Tu or vehicle by i.p.^27, 28^, prepared lysates from the brains, and assessed this pathway. Analysis of the lysates from several brain regions showed that Tu increases the level of LAMP2A and Bip and reduces the level of MEF2D in the striatum (Fig. 8a), consistent with the reports that striatal neurons exhibit special sensitivity to ER stress^29^. We focused on striatum tissue for the subsequent studies. Analysis of the lysosomes purified from the striatum showed that Tu induces a dose-dependent increase in the levels of phosphorylated p38α MAPK and phosphorylated LAMP2A (Fig. 8b). Co-administration of SB203580 to mice reversed Tu-induced increase in total and phosphorylated LAMP2A (Fig. 8c) and CMA activity (Fig. 8d).

**Fig. 9:**
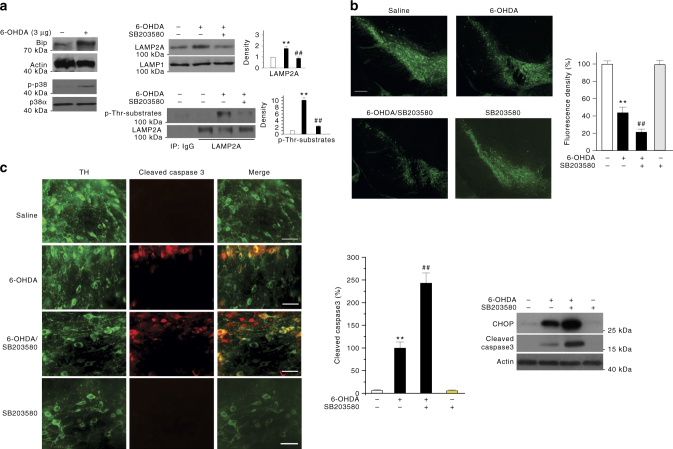
Neurotoxin engages ER-p38 MAPK–CMA in mouse brain in a model of PD. **a** 6-OHDA treatment engages ER-p38 MAPK–CMA pathway in mouse brain. Mice were administrated with 6-OHDA (3 μg) with or without co-injection of SB203580 (2 μg) by unilateral stereotaxic injection to the SN region and killed after 2 days (*n* = 16). Pooled SN tissues were analyzed for Bip, p-p38α MAPK, and p38α MAPK with the tissue lysate, and for LAMP2A and p-LAMP2A with the purified lysosomes. **b** Inhibition of p38 MAPK activity exacerbates 6-OHDA-induced loss of TH (tyrosine hydroxylase) positive neurons (green) in mouse SNc. Mice were stereotaxically injected with saline, 6-OHDA, 6-OHDA plus SB203580, and SB203580 at dosages as described in **a**. Mouse brain SNc slices were immunostained for TH (scale bar = 200 µm). Right panel shows the quantification of TH positive neurons in each group. **c** Inhibition of p38 MAPK exacerbates 6-OHDA-induced activation of caspase 3. The SNc slices from mice treated as described in **b** were immunostained for TH (green) and activated caspase 3 (red) (scale bar = 25 µm). Middle panel shows the quantification, and right panel shows the level of CHOP and cleaved caspase 3 by western blot. Quantifications are shown for **a**, **b**, and **c** (*n* = 6 per group. All values are mean ± s.d. (ANOVA with Turkey test. **p < 0.005 vs. control and ^##^ *p* < 0.005 vs. 6-OHDA)

Dysfunction of ER and lysosomes has been implicated in the pathogenic process of PD^30, 31^. We tested ER-p38 MAPK–LAMP2A–CMA in models of PD in culture and in vivo. For cellular model, we treated SN4741 cells with 6-OHDA, a neurotoxin widely used to model PD and shown to induce CMA in SN4741 cells^18^. 6-OHDA treatment induced Bip, reduced MEF2D, and activated p38 MAPK in the cellular lysate, increased total/phosphorylated LAMP2A and its oligomerization in purified lysosomes, and stimulated CMA activity (Supplementary Fig. 7a–c). In contrast, inhibition of p38 MAPK by SB203580 significantly attenuated these effects of 6-OHDA, suggesting that 6-OHDA engages ER-p38 MAPK–CMA pathway. Next, we tested the role of ER-p38 MAPK–CMA in a 6-OHDA-induced mouse model of PD^32^. We stereotaxically injected 6-OHDA unilaterally into one side of mouse striatum and used the contralateral side as a control. Analysis of the SN region showed that 6-OHDA increases the levels of Bip, p-p38 MAPK, and total as well as phosphorylated LAMP2A (Fig. 9a). Co-administration of SB203580, which effectively blocked p38α MAPK activity in vivo as it reversed 6-OHDA-induced phosphorylation of p38 MAPK substrate ATF2 (Supplementary Fig. 8), attenuated 6-OHDA-induced increase in total and Thr phosphorylated lysosomal LAMP2A in the SN (Fig. 9a, top right). Immunohistochemical analysis of the mouse brain revealed that 6-OHDA causes a significant loss of tyrosine hydroxylase-positive (TH^+^) neurons in the SNc. The loss of DA neuronal viability was significantly exacerbated by co-administration of SB203580 (Fig. 9b). Similarly, SB203580 also markedly enhanced the level of cleaved caspase 3 and CHOP following 6-OHDA (Fig. 9c). Since SB203580 alone did not affect level of TH, cleaved caspase 3, and CHOP in mice (Fig. 9b, c), these data support the interpretation that ER-p38 MAPK–CMA pathway protects the SNc DA neurons against 6-OHDA-induced toxicity in vivo.

## Discussion

Our studies have revealed that ER and lysosomes, the two key cellular stress organelles, are functionally coupled via p38 MAPK, the most critical stress signal transducer^21, 33, 34^, via a process that we have termed ERICA. The mechanism underlying this link involves PERK-dependent activation and lysosomal association of MKK4, specific activation of a pool of lysosomal p38 MAPK, direct phosphorylation of LAMP2A by p38 MAPK, and phosphorylation-dependent increase and conformational change of LAMP2A on the lysosomal membrane, and activation of CMA process (Supplementary Fig. 9). Our findings establish further that engaging ERICA constitutes a critical protective response for maintaining cellular homeostasis and viability under ER stress. At least part of the mechanism of protection involves CHOP, which appears to be downstream of and inhibited by CMA following activation of ERICA. Our data indicate that all the ER stressors that we tested under our experimental conditions trigger ERICA. This unexpected tight coupling between ER/UPR and CMA suggests that ER stress response and CMA are highly coordinated processes, making CMA a critical and integral component or extension of the machinery that responds to ER stress. This raises interesting and fundamental questions of whether recruiting CMA is obligatory for cells to handle ER stress properly and whether other non-UPR ER signals may also engage this pathway under different conditions. Answers to these questions will help establish the role of ERICA in a broader range of cellular processes and responses.

The initial UPR is widely considered to be cellular protective^3^. Based on our data, we believe and conclude that at least a critical part of this protection is mediated through the activation of CMA, establishing CMA as a major protective component engaged by ER in response to stress. However, CMA is not the only survival mechanism under ER stress. It is likely that depending on the nature of stress, cells activate CMA and/or other varying mechanisms for protection^35, 36^. The precise mechanisms by which CMA mediates cellular protection may vary and depend on specific cellular context and require further investigation. This could involve maintaining the function of key survival factors such as MEF2D^12^ or global homeostasis of long-lived proteins^37, 38^. Both ER stress and loss of CMA function have been implicated in the pathogenic process of PD^2, 39^. We showed that in 6-OHDA model of PD, ERICA may play a key role in protecting the SNc DA neurons from neurotoxin-induced death. Therefore, it is possible that dysfunction of this pathway may contribute to the pathogenic process of PD. This is highly consistent with recent findings showing that CMA helps cells maintain homeostasis and the loss of its activity is associated with PD^8, 39^. Given that a wide spectrum of signals can trigger ER stress, it is likely that ERICA may also be involved in response to various other physiological and pathological stresses, and play a role in other neurodegenerative or non-neurological diseases. One recent study shows that chemical activators of PERK have high therapeutic potential for ER stress-associated diseases^40^, supporting the possibility that targeting ERICA may be a promising neuroprotective strategy for neurodegenerative diseases.

One of the intriguing findings of our study is that ER stress induces a robust activation of a pool of MKK4 and p38 MAPK associated directly with lysosomal membrane. p38 MAPK has been observed to localize to the nucleus, cytosol, and mitochondria^41, 42^, but has not been reported to be associated with lysosomes previously. Our findings broaden the subcellular location where MKK4 and p38 MAPK function and raise the possibility that signals not directly initiated from ER may engage the lysosomal MKK4 and p38 MAPK to regulate CMA. The pending question is how PERK activates and drives MKK4 to lysosomes under ER stress. PERK is a transmembrane protein kinase residing in the ER membrane. Its well-known role during ER stress is to phosphorylate the alpha subunit of eukaryotic translation initiation factor 2, inactivating it, and leading to a rapid reduction of translational initiation, which helps the cells restore ER homeostasis^43^. Our findings show that PERK is required for ERICA, and thus establishes the regulation of CMA as an additional critical function of PERK during ER stress. Our data indicate that PERK engages CMA via MKK4. PERK may modulate MKK4 activity either directly or indirectly. For example, although our data suggest that ASK1 is not required ERICA, it is possible that PERK may regulate the other known upstream MAPKKKs to activate MKK4. Alternatively, PERK may activate MKK4 by direct interaction. These different possibilities are being actively investigated.

Our findings suggest that phosphorylation at T211 and T213 constitutes a key regulatory event in modulating LAMP2A oligomerization and level on the lysosomal membrane. This is the first example showing that signal-mediated post-translational modification of LAMP2A modulates its function. How these dual phosphorylation events increase LAMP2A level at the lysosomal membrane and promote the receptor to assume a more active oligomeric conformation is not known. Since these two sites are in the lysosomal luminal domain of the protein, topologically, how membrane-associated p38 MAPK engages the target sites also remains unclear. Given that the level and conformation of LAMP2A on the lysosomal membrane is quite dynamic and modulated by multiple factors^39, 44^, it would require a comprehensive assessment of the full life cycle of LAMP2A to determine the precise steps at which phosphorylation of T211 and 213 impacts LAMP2A.

## Methods

### Chemicals

The chemicals and their sources are as follows: SB203580 (cat#5633) from Cell Signaling; 4μ8C (IRE1 inhibitor III, cat#412512), cycloheximide (cat#239763), geldanamycin (cat#345805), and GSK2606414 (PERK inhibitor I, cat#516535) from EMD Millipore; ^32^P-ATP (cat#50-905-0808) from Perkin Elmer; 3-methyladenine (3MA, cat#M9281), 6-aminonicotinamide (6-AN, cat#A68203), 6-hydroxydopamine (6-OHDA, cat#H4381), ammonium chloride (NH_4_Cl, Cat#254134), ATP (cat#A2383), BFA (#B7651), DAPI (cat#D9542), MG132 (cat#M7449), Tg (cat#T9033), Tu (cat#654380), and β-mercaptoethanol (cat#M6250) from Sigma-Aldrich; and Hoechst 33258 (cat#H3569) from Thermo Fisher.

### Antibodies

The following primary antibodies were used at the dilution of 1:1000 for IB and 1:100 for IF except specific ones indicated. The secondary antibodies were used at the dilution of 1:10000 for IB and 1:500 for IF. The antibodies and their sources are as follows: antibodies to ATF6 (cat#ab37149), LAMP2A (cat#ab18528, cat#ab125068), LAMP2B (cat#ab18529), Hsc70 (cat#ab2788), and ribonuclease A antibody (cat#ab94417, 1:2000) from Abcam; antibodies to c-raf (cat#610151), MEF2D (Cat#610775), and p-p38a MAPK (cat#612288) from BD Biosciences; antibodies to ATF2 (cat#9226), bip (cat#3183), caspase 3 (cat#9662), cathepsin B (cat#31718), cathepsin D (cat#2284), CD63 (cat#55051), CHOP (cat#2895), cleaved caspase 3 (cat#9664), cytochrome c (cat#12963), GAPDH (cat#5174), IRE1α (cat#3294), LAMP1 (cat#9091), LC3A/B (cat#4108), MKK4 (cat#9152), myc (cat#2276), p38 MAPK (cat#8690), PARP (ca#9532), p-ATF2 (cat#9225), PDI (cat#3501), PERK (cat#3192), p-MKK4 (cat#9156), p-ser-MAPK/CDK substrates (cat#2325), p-threonine (cat#9391), and tyrosine hydroxylase (TH, cat#2792) from Cell Signaling; antibodies to actin (cat#A5441, 1:5000), secondary antibodies anti-rabbit (cat#A0545), and anti-mouse (cat#A8924) from Sigma; antibodies to p-IRE1a (cat#PA1-16927) and p-PERK(MA5-15033) from Thermo Fisher; antibody to LAMP2C (1:500), a gift from Dr. Tomohiro Kabuta (National Center of Neurology and Psychiatry, Tokyo Japan); and mouse IgG (Cat#015-000-003) and rabbit IgG (cat#011-000-003) from Jackson ImmunoResearch. The uncropped scans are shown in Supplementary Figs. 10 and 11.

### Plasmids

p38α MAPK-Flag and DN-p38α MAPK-Flag were gifts from Dr. Jiahuai Han (Xiamen University, China); pCMV6-CHOP was a gift from Dr. Shi-Yong Sun (Emory University Winship Cancer Institute); pcDNA/Hygro-anti-sense-Lamp2a was a gift from Janice S. Blum (Indiana University School of Medicine, Indianapolis); wt-LAMP2A (mouse) was kindly provided by Dr. Ana Maria Cuervo (Albert Einstein College of Medicine); and wt-LAMP2A (human, cat#RC221216) was purchased from OriGene. Mt (T211A/T213A)-LAMP2A and mt (T211E/T213D)-LAMP2A were generated from wt-LAMP2A with primers F: 5′-TGCCATCTC CTACTACAGCACCTGCTCCAAAGGAAAAACCA-3′ and R: 5′-TGGTTTTTCCTTTGGAG CAGGTGCTGTAGTAGGAGATGGCA-3′ and F: 5′-CTTCTGGTTTTTCCTTTGGATCAGGTTC TGTAGTAGGAGATGGCACAGTGGTGTG-3′ and R: 5′-CACACCACTGTGCCATCTCCTA CTACAGAACCTGATCCAAAGGAAAAACCAGAAG-3′, respectively, with QuickChange II XL Site-Directed Mutagenesis kit (cat#200522, Agilent).

### siRNAs

All products including ASK1 (cat#sc-29749), ATF6 (cat#sc-45950), CHOP (cat#sc-35438), control (cat#sc-37007), IRE1 (cat#sc-40706), MKK3 (cat#sc-35908), MKK4 (cat#sc-35910), MKK6 (cat#sc-35914), and PERK (cat#sc-36214) were purchased from Santa Cruz.

### Cell culture

SN4741 cells (a gift from Dr. Jin H. Son, Cornell University Medical College) were cultured as described previously^12^. Briefly, the cells were grown in DMEM supplemented with 10% fetal bovine serum, 0.6% d-glucose, 1.4 mM l-glutamine, and 50 U/ml penicillin/streptomycin at 33 °C and 5% CO_2_. The cells were split when they reached 70–80% confluence. HEK293T cells (ATCC® CRL-3216™) were cultured in regular DMEM complete medium at 37 °C and 5% CO_2_.

### Isolation and subfractionation of lysosomes

Lysosomes were purified from SN4741 cells using a lysosome isolation kit from Sigma (Cat# LYSISO1). Briefly, 19% Optiprep fraction was loaded on the iodixanol gradient and ultracentrifuged at 150,000 × *g* for 4 h. The intactness of the lysosomes was evaluated by Neutral Red reagent according to the manufacturer’s instruction. The lysosomes from mouse brain were isolated from a light mitochondrion-lysosome fraction (LMF) using Percoll gradients^45, 46^. Briefly, 0.5 ml suspended LMF was layered onto 4 ml of the 24% Percoll solution and centrifuged at 62,500 × *g* in a Beckman SW40.1 rotor for 2 h. The lysosome band is close to the bottom of the gradient. Lysosomal matrices and membranes were separated according to the procedure described previously^47, 48^. Briefly, lysosomes disrupted by hypotonic shock (0.025 M sucrose, incubated for 30 min on ice) were subjected to ultracentrifuge at 105,000 × *g* for 1 h using Beckman TLA 120.1 rotor. The supernatants were lysosomal matrices. The pelleted membranes were washed once with MOPS buffer [10 mM 3-(N-morpholino) propanesulfonic acid, 0.3 M sucrose, pH 7.3] and re-centrifuged at 105,000 × *g* for 1 h.

### Lysosome binding and uptake assays

CMA activity was evaluated by transport of CMA substrates RNaseA (Sigma, Cat#: R-5500) or MEF2D into isolated lysosomes as previously described^14, 15, 49, 50^. Freshly purified lysosomes treated with or without chymostatin (100 μM, 10 min on ice) were co-incubated with in vitro purified RNase A or cellular lysates with overexpressed Flag-MEF2D in modified MOPS buffer (with additional 10 mM ATP and 5 μg/ml Hsc70 peptide) for 20 min at 37 °C. In uptake assay, treatment with proteinase K in MOPS buffer after incubation was necessary. Lysosome pellets were subjected to SDS-PAGE and western blotting after being washed four times with cold PBS and centrifuged at 21,000 × *g* for 10 min at 4 °C.

### MTT and WST-1 assays

Cell survival was measured by MTT assay with the kit (Cat# 11465007001, Sigma-Aldrich) and following procedures provided by the manufacturer. Briefly, the cells were grown in a 24-well plate. After treatment, 50 µl of MTT reagent was added to each well and incubated for 3 h. The plate was then allowed to stand overnight in the incubator with 500 µl solubilization buffer. Absorbance at 580 and 670 nm was measured on a Bio-Tek plate reader. For WST-1 (cat#11644807001, Sigma-Aldrich) assay, the cells were incubated with 50 µl of WST-1 solution for 2 h in 24-well plates. Cell viability was measured at 450 and 600 nm in a microplate reader. For both MTT and WST-1 assays, data were expressed as mean ± s.e.m. of corresponding controls from three independent experiments, 6 repeats per each treatment of one independent experiment. The SPSS one-way ANOVA was employed to analyze all data with the post hoc Turkey to compare the difference between any two groups.

### Native gel electrophoresis

Lysosomal complexes were subjected to continuous native polyacrylamide gel electrophoresis (native-PAGE) with no boiling in non-reducing loading buffer as Tomashek et al. described^51^. Native PAGE was carried out using 6% polyacrylamide continuous gels in the absence of SDS. The LAMP2A-containing complexes were detected using anti-LAMP2A antibody or myc antibody.

### In vitro kinase assay

Purified endogenous LAMP2A, wt-LAMP2A, mt(A/A)-LAMP2A, or mt(E/D)-LAMP2A proteins were incubated with the active p38α MAPK (cat#14-687-K, EMD Millipore) in a kinase buffer (containing 20 μM ATP and 10 μCi of γ-^32^P-ATP) for 30 min at 30 °C.

### RT-PCR and quantitative real-time PCR

Total RNAs were extracted using TRIzol reagent (Invitrogen). After DNase treatment (Ambion, Cat#: AM1906), 2 μg RNA was transcribed using RETROscript First Strand Synthesis kit (Ambion, Cat#AM1710). For XBP1 splicing, 5 µl RT product was applied for PCR program (94 °C 4 min, (35 cycles of 94 °C 10 s, 65 °C 30 s, and 72 °C 30 s) and 72 °C 10 min) with primers for mouse XBP1 (F 5′-AAACAGAGTAGCAGCG CAGACTGC-3′ and R 5′-TCCTTCTGGGTAGACCTCTGGGAG-3′)^52^ and primers for actin control (F 5′-GGGTCAGAAGGATTCCTATG-3′ and R 5′-GGTCTCAAA CATGATCTGGG-3′). For LAMP2A and MEF2D, the amplification was carried out on Mx3000 P (Agilent) using SYBRGreen qPCR Master Mix (Agilent, Cat#600882) as following: 95 °C, 3 min; followed by 40 cycles of 95 °C, 10 s; 60 °C, 20 s. The target genes and primers were: LAMP2A (F 5′-GTCTCAAGCGCCATCATACT-3′ and R 5′-TCCAAGGAGTCTGTCTTAAGTAGC-3′); MEF2D (F 5′-GTCCCCGTTTCTCTCAGCAA-3′ and R 5′-CTTGATGCTGATGTGGGGGT-3′); and actin (F 5′-AAGGACTCCTATAGTGGGTGACGA-3′ and R 5′-ATCTTCTCCATGTCGT CCCAGTTG-3′)^53, 54^.

### In vivo experiments

All procedures were approved by the IACUC of Emory University.To create in vivo ER stress mouse model, 16-week-old male C57BL/6 mice were assigned to four experimental groups (four animals per group; based on the means and standard deviations of HT positive neurons in control and treated mice, we calculated animal numbers using the power analysis at http://www.biomath.info/power/ttest.htm, *α* = 0.05, power = 1−*β* = 0.8) by the block randomization method^55^. Tu (1 μg/g body weight), SB203580 (2 μg/g body weight), or isotonic saline solution (volume equal to that of drugs) were injected intraperitoneally (i.p.) into 16-week-old male C57BL/6 mice (Jackson Laboratory). The mice were killed 1 day or 2 days after injection for biochemical assessment. 6-OHDA lesion mouse model was created as described. Stereotaxic surgery procedures: 8-week-old male C57BL/6 mice (Jackson Laboratory, weighing ~20 g) were placed in a stereotaxic device under 1.5% isoflurane anesthesia. 6-OHDA (3 μg) and SB203580 (2 μg) were injected into the left substantia nigra pars compacta (SNpc) at a rate of 0.1 μl/min. The needle was left in place for 5 min after the injection before retraction. The injection was performed using a Hamilton syringe at the following coordinates: AP: −1.2 mm, ML: −1.1 mm, and DV: −5 mm relative to Bregma according to the Mouse Brain Atlas in Stereotaxic Coordinates. The control received the same amount of saline only. The mice were killed after 2 days following injection. Brains were removed for biochemical or histological assessments.

### Statistical analysis

All data were shown as mean ± s.d. or mean ± s.e.m., and comparisons were made by unpaired two-tailed *t* test for two groups and the one-way ANOVA with Tukey post hoc analysis for multiple groups. *p*-value < 0.05 was considered statistically significant.

### Data availability

The authors declare that all data supporting the findings of the study are contained within the article and its supplementary files.

## Electronic supplementary material

Supplementary Information
