# Supplementary material for: Phosphorylation of LAMP2A by p38 MAPK couples ER stress to chaperone-mediated autophagy
Source: Nat Commun. 2017 Nov 24;8:1763. doi: 10.1038/s41467-017-01609-x (PMC5701254; doi:10.1038/s41467-017-01609-x)
Supplement: Supplementary file 1 — Supplementary Information [file 41467_2017_1609_MOESM1_ESM.docx]

**
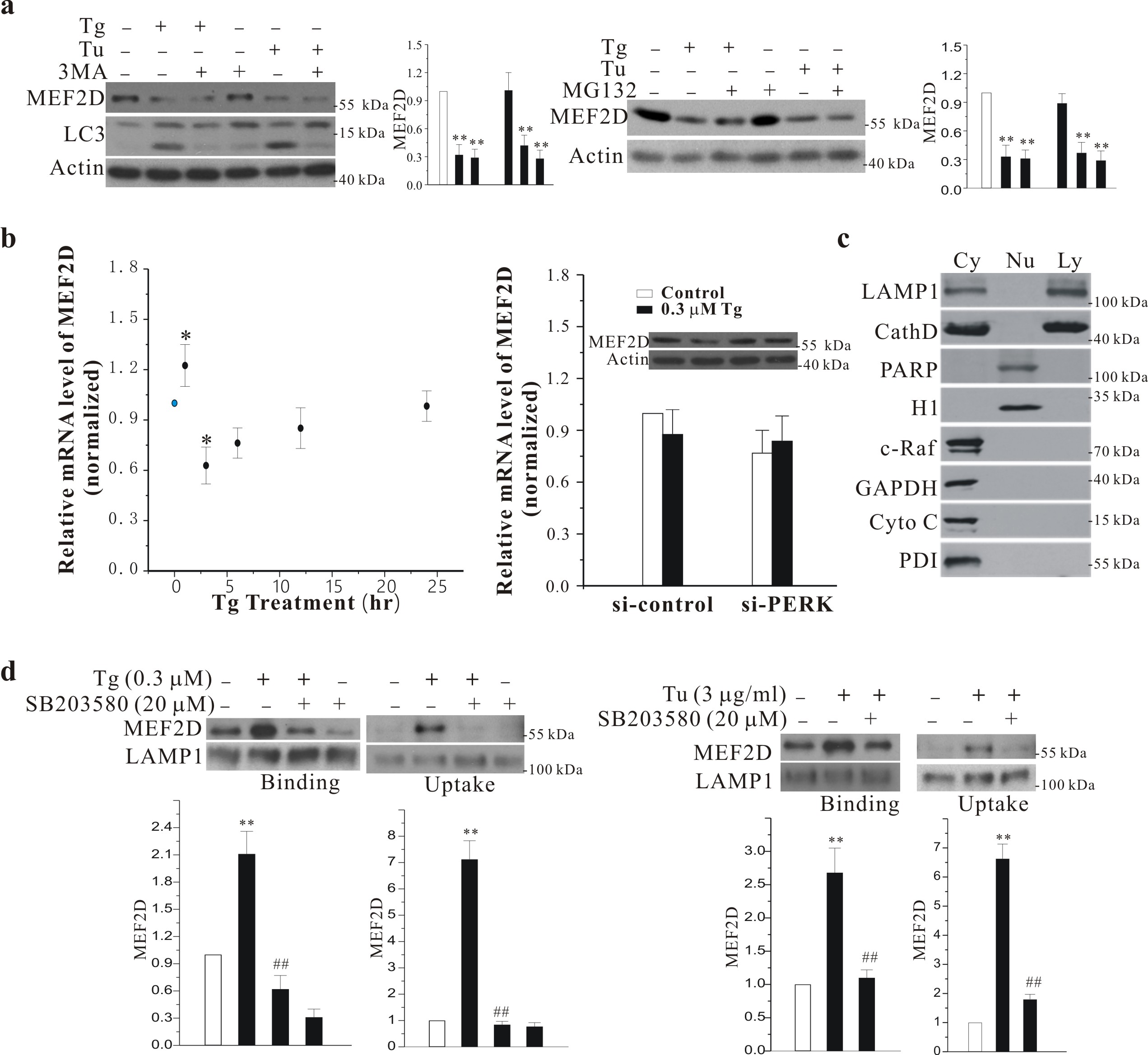
**

**Supplementary Figure 1 | ER stress activates CMA by increasing key CMA regulator LAMP2A in lysosomes**. (**a**) The effect of 3-MA and MG132 on MEF2D level. SN4741 cells were exposed to Tg (0.3 μM) and Tu (0.3 μg/ml) for 12 h with or without 30 min pretreatment of 3-MA (10 mM, left panel) or MG132 (10 μM, right panel). Total proteins were collected for western blot with MEF2D and actin antibodies. Middle left panel also shows the function of 3-MA with LC3 conversion. (**b**) The effect of knockdown of PERK on the level of MEF2D mRNA. SN4741 cells were treated with Tg (0.3 μM) for different times (left panel) or transfected with si-Control or si-PERK RNAs for 60 h and then treated with Tg (0.3 μM) for 12 h (right panel). The levels of MEF2D mRNA in SN4741 cells were determined by qRT-PCR. Data are shown as mean ± s.d [n=3×4 (one-way ANOVA with Turkey test), *P<0.05 vs control]. The inset shows the level of MEF2D under the same condition. (**c**) The purity of lysosome isolated from SN4741 cells (n=3). LAMP1 and Cathepsin D (Cath D) are lysosome markers; PDI is an ER marker; Cytochrome C (Cyto C) is a mitochondrial marker; c-Raf and GAPDH are cytoplasmic (Cy) markers; PARP and histone H1 are nuclear (Nu) markers. (**d**) ER stress-induced CMA activation. SN4741 cells after exposure to Tg with or without SB203580 for 12 h. Purified lysosomes from samples were analyzed for binding and uptake assay with MEF2D as a substrate of CMA. Quantifications for panels (a) and (d) are shown [n=3. All values are s.d. (ANOVA with Turkey). **p<0.005 vs. control and ^##^p<0.005 vs. Tg or Tu challenge alone].


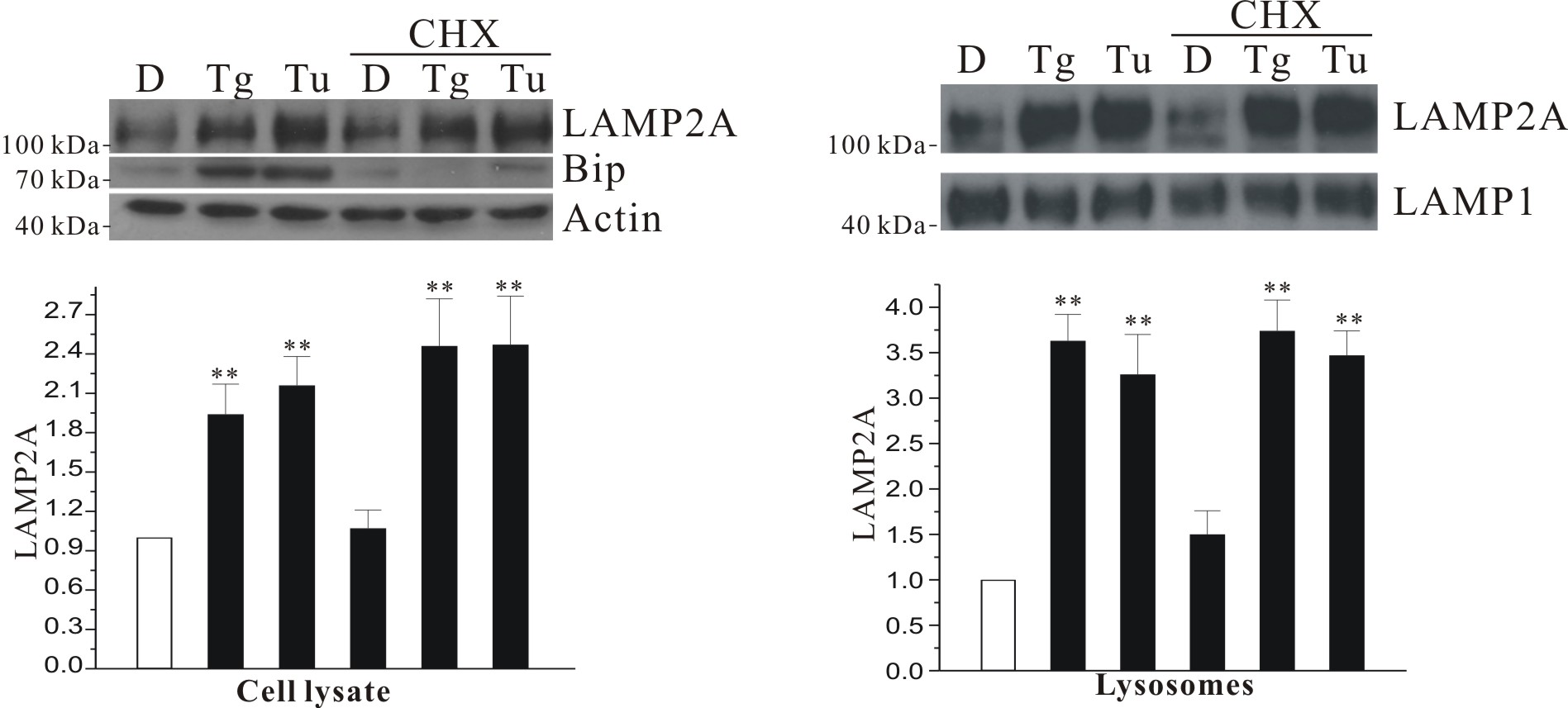


**Supplementary Figure 2 |** **ER stress increases LAMP2A independent of new protein synthesis.** ER stress induces the CMA activation independent of new protein synthesis. SN4741 cells were treated with DMSO, Tg (0.3 μM), and Tu (3 μg/ml) in the presence or absence of cycloheximide (CHX, 50 μg/ml) for 12 h. Total proteins (left) or purified lysosomes (right) were blotted as indicated. Bottom panels show quantification of LAMP2A [n=3. All values are mean ± s.d. (ANOVA with Turkey). **p<0.005 vs. control].

**
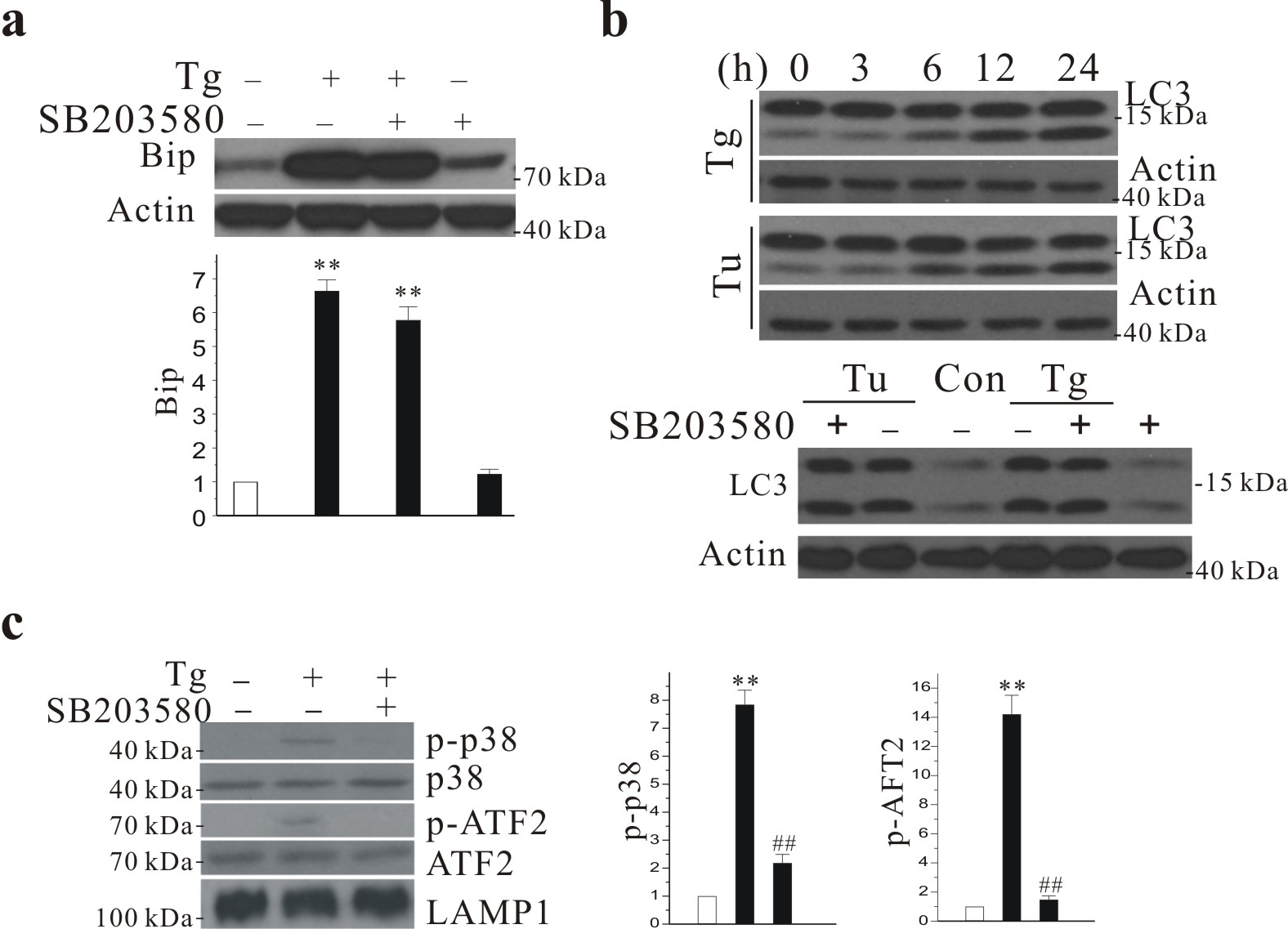
**

**Supplementary Figure 3 | p38 MAPK mediates ER stress-induced activation of CMA.** (**a**) The effects of SB203580 on Bip induced by Tg. SN4741 cells were exposed to Tg with or without SB203580 for 12h. Total proteins were blotted for Bip and actin. (**b**) The effect of SB203580 on the activation of macroautophagy induced by ER stress. SN4741 cells were treated with Tg (0.3 μM; top) or Tu (3 μg/ml; middle) for 0 to 24 h in the presence or absence of SB203580 (20 μM) for 12 h. Total proteins were blotted for LC3 (bottom). (**c**) ER stress-induced activation of lysosomal p38 MAPK. SN4741 cells were exposed to Tg (0.3 μM) for 2 h with or without SB203580. Purified lysosomes were blotted for various proteins as indicated. Quantifications for panel (a) and (c) are shown [n=3. All values are mean ± s.d. (ANOVA with Turkey). **p<0.005 vs. control and ^##^p<0.005 vs Tg].


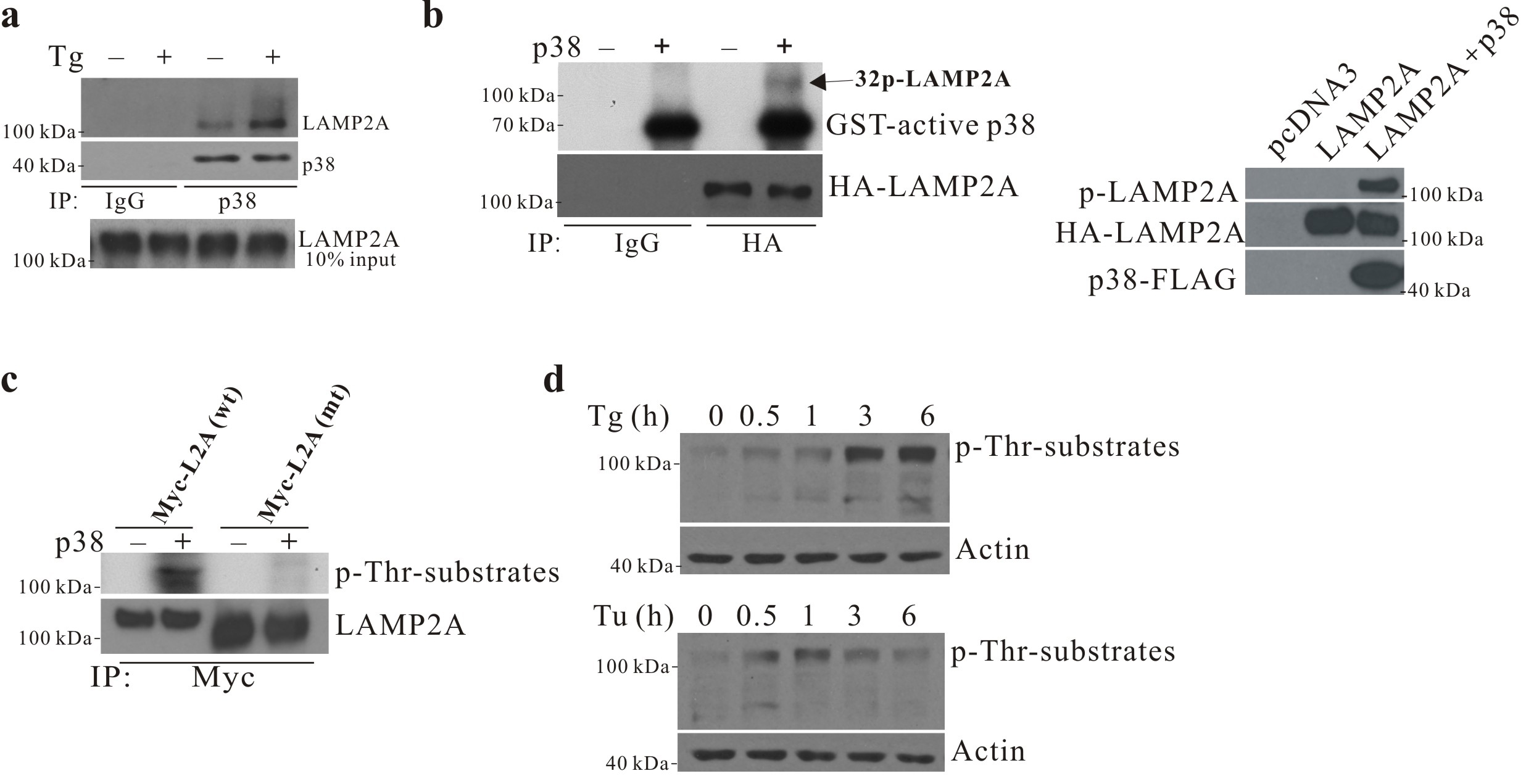


**Supplementary Figure 4 | p38** MAPK **interacts with and phosphorylates LAMP2A.** (**a**) Interaction between p38 MAPK and LAMP2A. SN4741 cells were treated with or without (0.3 μM) for 2 h. Total proteins were collected for Co-IP with an anti-p38 MAPK antibody and IB with an anti-LAMP2A antibody. (**b**) Phosphorylation of LAMP2A by p38 MAPK. HEK293 cells were transfected with HA-LAMP2A (cloned from mouse *lamp2a* mRNA) for 40 h and proteins were collected for IP with an anti-HA antibody or IgG. The precipitates were incubated with purified p38 MAPK *in vitro* kinase assay with ^32^P-ATP (left). HEK293 cells were co-transfected with HA-LAMP2A and p38 MAPK for 40 h, and total proteins were collected for IP with an anti-HA antibody and IB with an anti-p-Thr substrate antibody (right). (**c**) Phosphorylation of LAMP2A at T211 and 213. HEK293 cells were transfected with wild type-LAMP2A or T211A/T213A mutant with or without p38 MAPK for 40 h and proteins were collected for IP with an anti-myc antibody and IB with an anti-p-Thr substrate antibody. (**d**) ER stress-induced phosphorylation of substrates at Thr residues. SN4741 cells were exposed to Tg (0.3 μM) or Tu (3 μg/ml) for the indicated periods of time. Total proteins were blotted with an anti-p-Thr-substrate antibody.


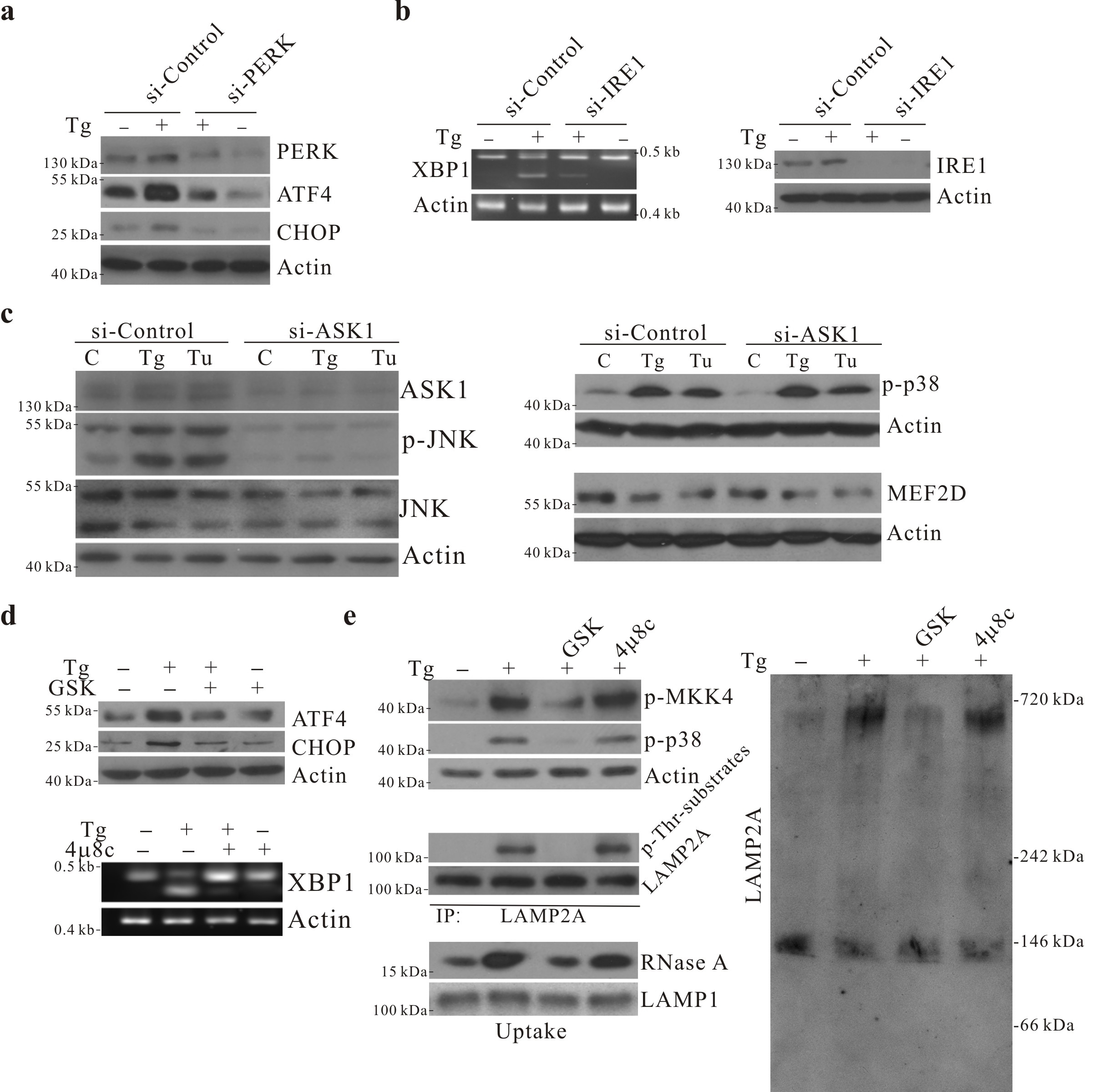


**Supplementary Figure 5 | PERK and MKK4 activate the p38 MAPK-CMA pathway under ER stress.** (**a**) Attenuation of ER stress-induced ATF4 and CHOP by PERK knockdown. SN4741 cells were transfected with si-Control or si-PERK RNAs for 60 h and then treated with Tg (0.3 μM) for 12 h. Total proteins were blotted for PERK, ATF4, CHOP, and actin. (**b**) The effect of IRE1 knockdown on Tg-induced XBP1 splicing. SN4741 cells were transfected with si-Control or si-IRE1 RNAs for 60 h and then treated with Tg (0.3 μM) for 12 h. Total RNAs were extracted for RT-PCR with primers for unspliced and spliced XBP1 RNA species (left). The efficiency of si-IRE1 was shown by western blot in the right panel. (**c**) The effect of ASK1 knockdown on ER stress-induced CMA activation. SN4741 cells were transfected with si-Control and si-ASK1 for 71 h, and exposed to Tg and Tu for 1 h. Total proteins were collected for western blot with ASK1, p-JNK, JNK, and actin antibodies (left). SN4741 cells were transfected for 70 h and exposed to Tg (0.3 μM) or Tu (3 μg/ml) for 2 h. Total proteins were collected for western blot with p-p38 MAPK antibody (top right). SN4741 cells were transfected for 60 h and exposed to Tg (0.3 μM) or Tu (3 μg/ml) for 12 h. Total proteins were blotted for MEF2D (bottom right). (**d** and **e**) The effect of inhibition of PERK on Tg-induced activation of CMA. SN4741 cells were treated with Tg in the presence or absence of GSK2606414 (10 μM) or 4µ8C (25 μM) for 12 h. Protein and RNA lysates were blotted for ATF4, CHOP (d, top), and XBP1 splicing (d, bottom), respectively. SN4741 cells were pretreated with GSK2606414 (10 μM) and 4µ8C (25 μM) for 1 h and then exposed to Tg (0.3 μM) for 2 h. Total proteins were blotted for p-MKK4 and p-p38 MAPK (e, top left), and for IP with LAMP2A and IB with p-thr-substrates antibody (e, middle left). Purified lysosomes from SN4741 cells with the above treatments except for Tg for 12 h were analyzed for uptake assay (e, bottom left) and for LAMP2A oligomerization (e, right).


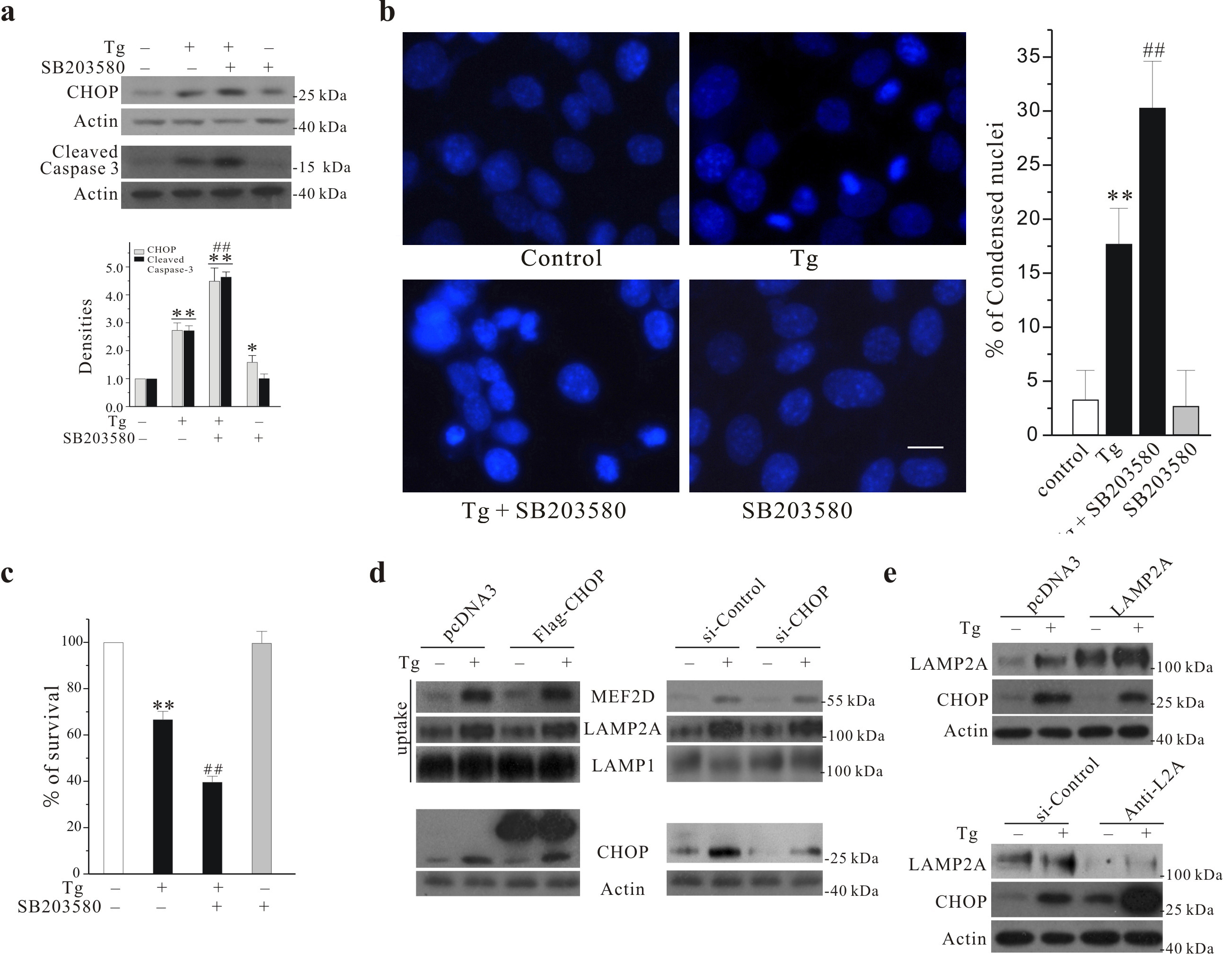


**Supplementary Figure 6 | ER stress-induced CMA is cellular protective.** (**a**) The effect of p38 MAPK inhibition on CHOP and cleaved caspase 3 following Tg treatment. SN4741 cells were exposed to Tg for 3 h and then incubated with SB203580 (10 μM) for 9 h or 21 h, and total proteins were blotted for CHOP or cleaved caspase 3, respectively. (**b**) The effect of inhibiting p38 MAPK on Tg-induced cell death. SN4741 cells were exposed to Tg (0.3 μM) with or without SB203580 added 3 h post Tg. The cells were stained with Hoechst 33258 after 24 h Tg treatment (scale bar=10 µm). The number of nuclei displaying nuclear fragmentation and condensation (at least 50% smaller than healthy control nuclei) is blindly counted as the dead cells. Right panel shows the statistical results of Hoechst staining, and the data are expressed as a percentage of condensed nuclei among the total nuclei (more than 300 per sample) from three independent experiments. (**c**) The effect of p38 MAPK inhibition on the viability of SN4741. SN4741 cells were exposed to Tg (0.3 μM) for 3 h and then incubated with SB203580 (10 μM) for 21 h. Cell viability was measured by WST1 assay. (**d**) The effect of altering CHOP level on LAMP2A and CMA activity following Tg treatment. SN4741 cells were transfected with pcDNA3 and CHOP for 24 h and then treated with Tg (0.3 μM) for 12 h, or transfected with si-Control and si-CHOP for 60 h, and treated with Tg for 12 h. Purified lysosomes were analyzed for uptake assay with MEF2D as CMA substrate and then re-probed with LAMP2A and LAMP1 antibodies. Bottom panels show the expressions of CHOP under each condition. (**e**) The effect of altering LAMP2A on CHOP following Tg treatment. SN4741 cells were transfected with pcDNA3 and LAMP2A for 24 h and then treated with Tg (0.3 μM) for 12 h, or transfected with si-Control and antisense LAMP2A (Anti-L2A) for 60 h and treated with Tg for 12 h. Total proteins were blotted for LAMP2A, CHOP, and Actin. Quantifications for panels (a), (b), and (c) are shown [n=3]. All values are mean ± s.d. (ANOVA with Turkey). *p<0.05 or **p<0.005 vs. control and ^##^p<0.005 vs. Tg alone.


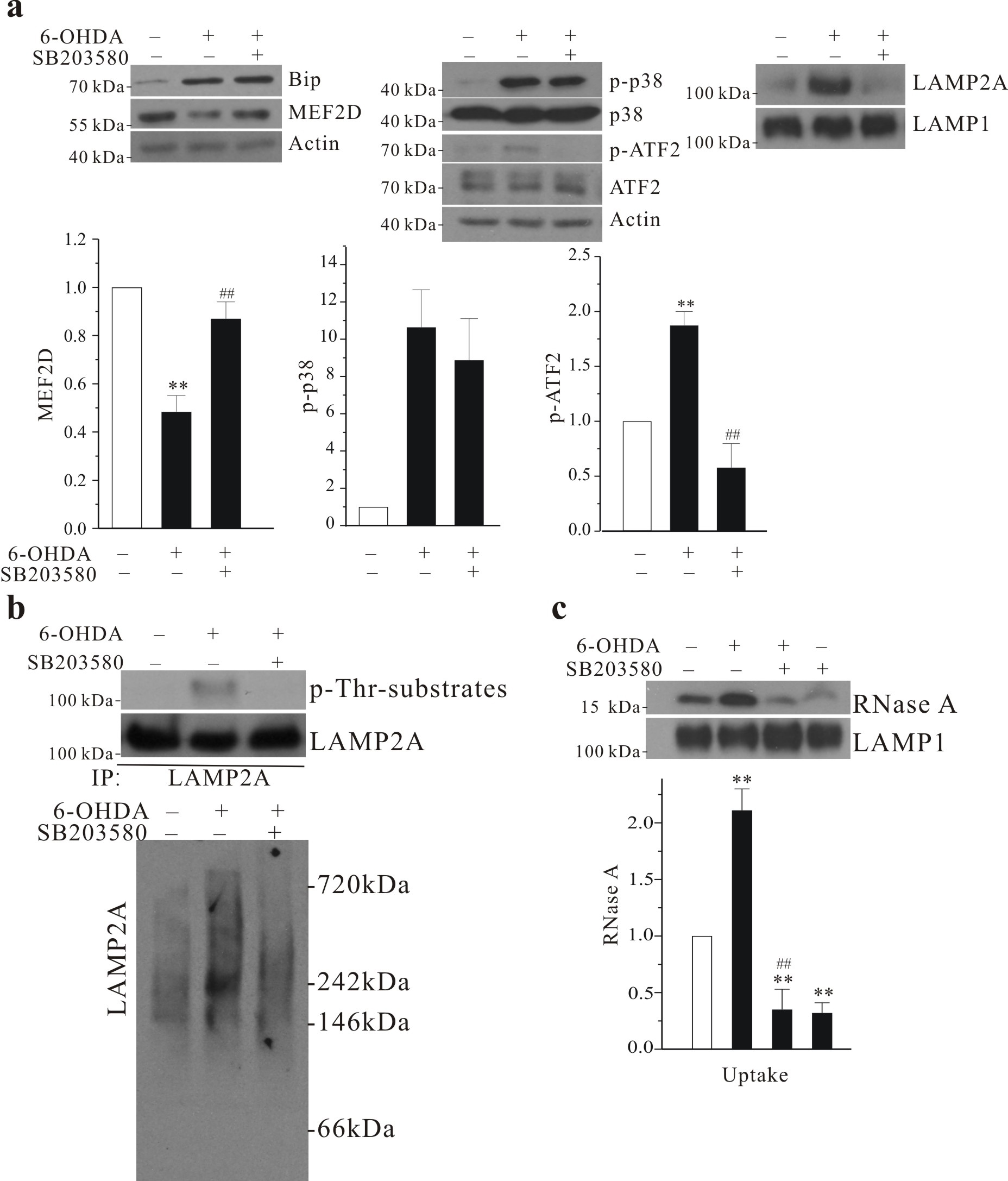


**Supplementary Figure 7 | 6-OHDA induces CMA in a p38** MAPK**-dependent manner**. (**a**) 6-OHDA-triggered activation of ER stress-p38 MAPK-CMA pathway in SN4741 cells. SN4741 cells exposed to 6-OHDA (40 μM) with or without SB203580 (10 μM) were analyzed for ER stress marker Bip and the CMA substrate MEF2D (top, 24 h post 6-OHDA), p-p38 MAPK, p38 MAPK, p-ATF2, and ATF2 (middle, 1 h post 6-OHDA), and LAMP2A in lysosomes (bottom, 12 h post 6-OHDA). Bottom graphs are quantifications. (**b**) 6-OHDA-induced activation of CMA in a p38-dependent manner. SN4741 cells were exposed to 6-OHDA (40 μM) with or without SB203580 (10 μM). After 2 h, samples were analyzed for LAMP2A phosphorylation (top), and after 12 h, samples were analyzed for LAMP2A oligomerization (bottom). (**c**) Inhibition of p38 by SB203580 blocks uptake activity of CMA induced by 6-OHDA. SN4741 cells after exposure to 6-OHDA (40 μM) with or without SB203580 (10 μM) for 12 h. Purified lysosomes from samples were analyzed for uptake assay. Bottom panels of (a) and (c) show quantification of MEF2D, p-p38, p-ATF2, and RNase A, n=3. All values are mean ± s.d. (one-way ANOVA with Turkey). **p<0.005 vs. control and ^##^p<0.005 vs. 6-OHDA.

**
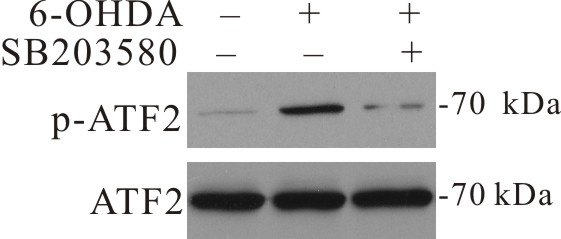
**

**Supplementary Figure 8 | Neurotoxin engages ER-p38 MAPK-CMA in mouse brain in a model of PD**. The effect of SB203580 on 6-OHDA-induced increase of p-ATF2 in mouse brain. Mice were administrated with 6-OHDA (3 μg) with or without co-injection of SB203580 (2 μg) by unilateral stereotaxic injection to the left SN region and sacrificed after 2 days. Proteins from the SN tissues were analyzed as indicated.

**
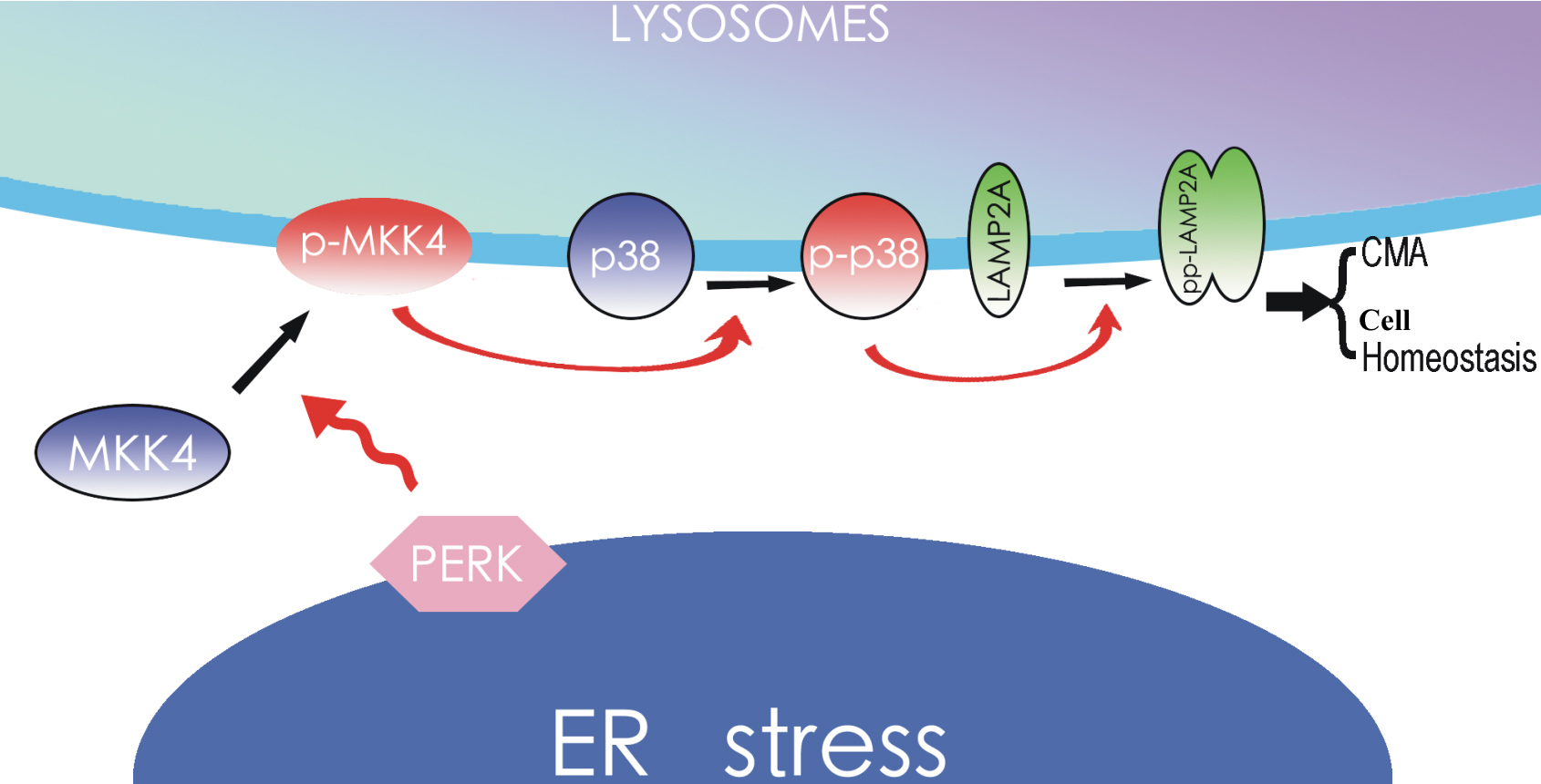
**

**Supplementary Figure 9 | Signal mechanisms underlying ER stress-induced chaperone-mediated autophagy (ERIC).** In the current study, we demonstrate that in response to ER stress, PERK is required for activating MKK4, and activated MKK4 (p-MKK4) translocates to the lysosomes, where it activates a lysosomal pool of p38 MAPK. The activated p38 MAPK in lysosomes directly phosphorylates LAMP2A at T211 and T213, driving its oligomerization and increasing CMA activity. This maintains cellular homeostasis and protects cells against ER stress.

**
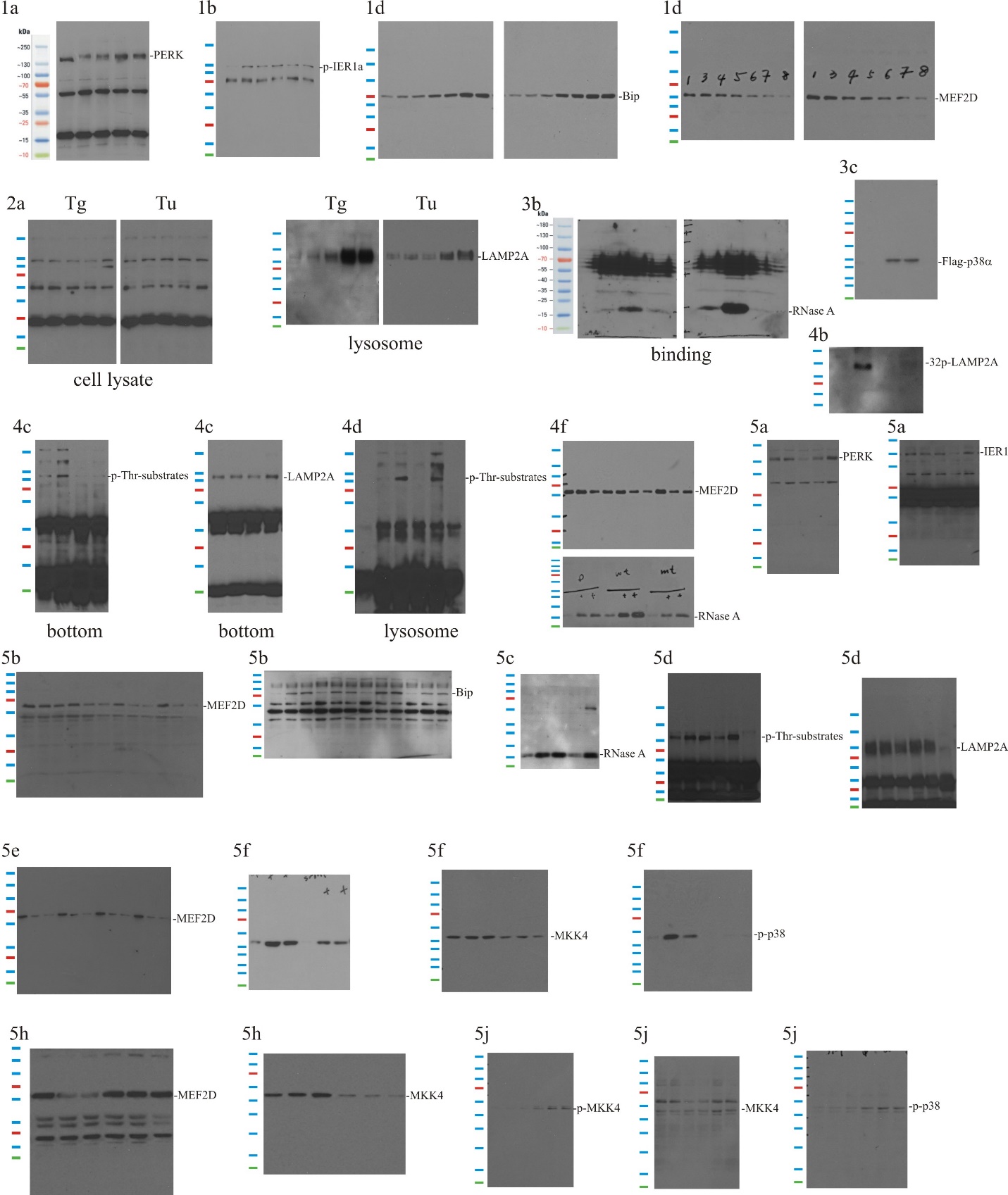
**

**Supplementary Figure 10 | Full blots are for main figures 1-9.** Both molecular weight markers are from Thermo Fisher with ranges 10 to 250 kda (Cat#26619) and 10 to 180 kDa (Cat#26616), respectively.

**
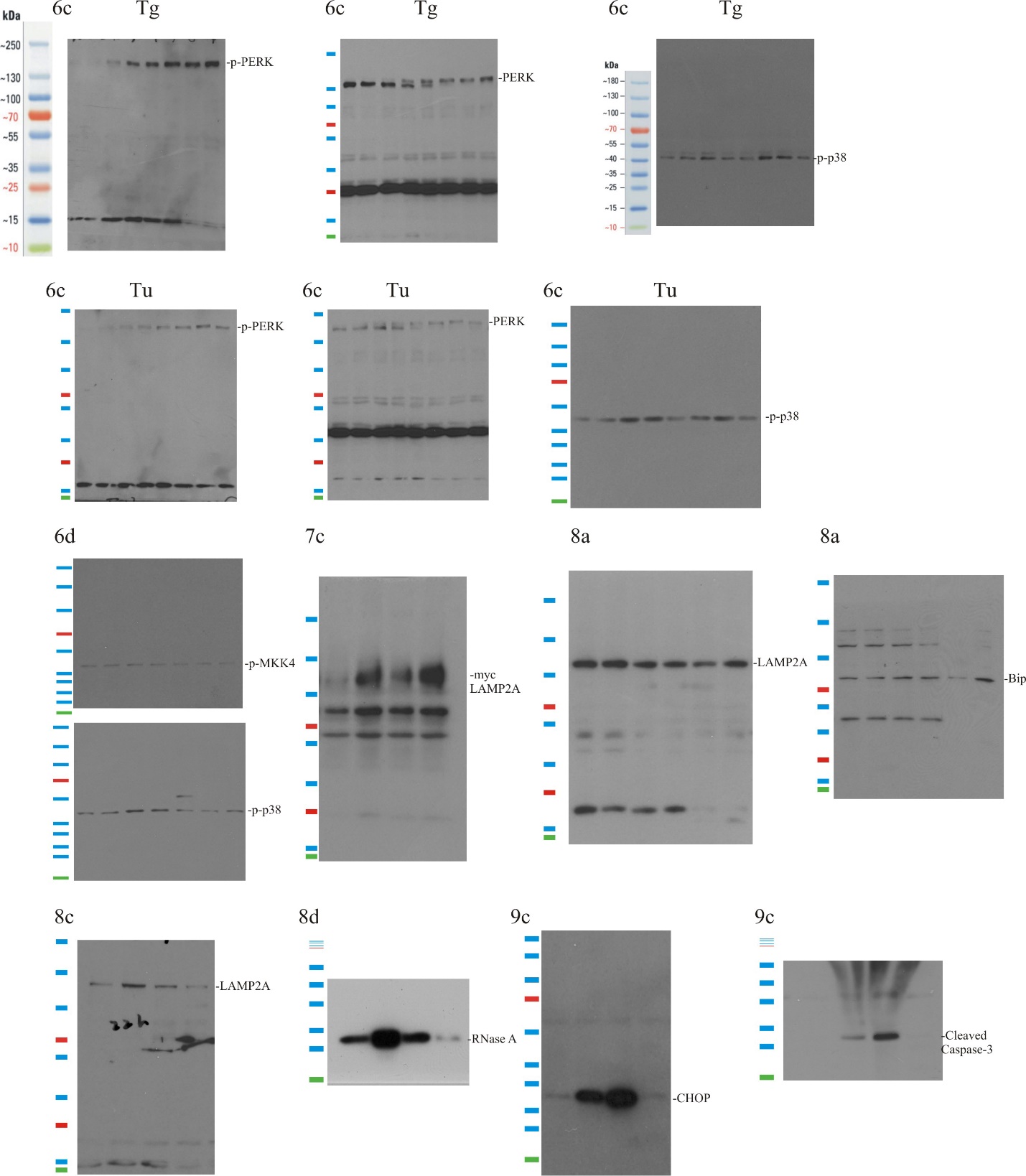
**

**Supplementary Figure 10 (cont) | Full blots are for main figures 1-9.** Both molecular weight markers are from Thermo Fisher with ranges 10 to 250 kda (Cat#26619) and 10 to 180 kDa (Cat#26616), respectively.

**
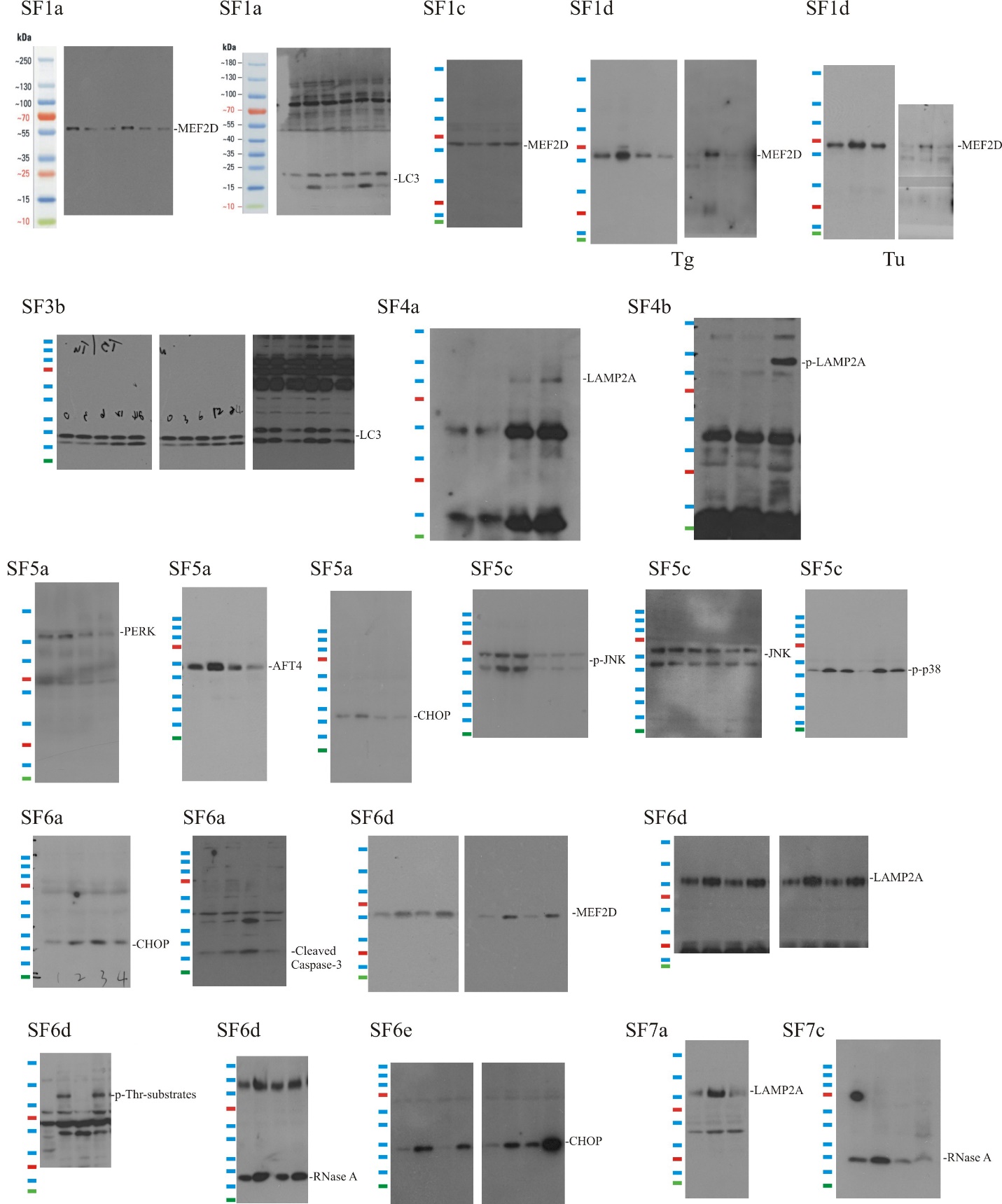
**

**Supplementary Figure 11 | Full blots are for Supplementary figures 1, 3, 4, 5, 6, & 7.** Both molecular weight markers are from Thermo Fisher with ranges 10 to 250 kda (Cat#26619) and 10 to 180 kDa (Cat#26616), respectively.

**Supplementary Table 1 | LAMP2A phosphorylation sites predicted for p38 MAPK**

GPS3.0 (Prediction of kinase-specific phosphorylation sites v3.0 from <http://gps.biocuckoo.org>) predicts the potential sites of human LAMP2A for p38 MAPK

**Position/Code/ Kinase/ Peptide Score/Cutoff**

207 S CMGC/MAPK/p38/MAPK14 TIHTTVPSPTTTPTP 3.892 3.189

211 T CMGC/MAPK/p38/MAPK14 TVPSPTTTPTPKEKP 5.855 3.189

213 T CMGC/MAPK/p38/MAPK14 PSPTTTPTPKEKPEA 5.181 3.189
